# An overview of the use of non-titanium MXenes for photothermal therapy and their combinatorial approaches for cancer treatment

**DOI:** 10.1039/d4na00931b

**Published:** 2024-12-26

**Authors:** Fathima Abdul Rahim, K. Niyas, Raju Vivek, Soyeb Pathan, P. Abdul Rasheed

**Affiliations:** a Department of Chemistry, Indian Institute of Technology Palakkad Kerala 678 623 India abdulrasheed@iitpkd.ac.in; b Department of Biological Sciences and Engineering, Indian Institute of Technology Palakkad Palakkad Kerala 678 623 India; c Bio-Nano Theranostic Research Laboratory, Cancer Research Program (CRP), School of Life Sciences, Bharathiar University Coimbatore Tamilnadu 641 046 India; d Research and Development Cell (RDC), Parul Institute of Applied Sciences, Parul University Vadodara Gujarat 391760 India; e Department of Chemistry, Parul Institute of Applied Sciences, Parul University Vadodara Gujarat 391760 India

## Abstract

Since the initial publication on the first Ti_3_C_2_T_*x*_ MXene in 2011, there has been a significant increase in the number of reports on applications of MXenes in various domains. MXenes have emerged as highly promising materials for various biomedical applications, including photothermal therapy (PTT), drug delivery, diagnostic imaging, and biosensing, owing to their fascinating conductivity, mechanical strength, biocompatibility and hydrophilicity. Through surface modification, MXenes can mitigate cytotoxicity, enhance biological stability, and improve histocompatibility, thereby enabling their potential use in *in vivo* biomedical applications. MXenes are also known for their ability to absorb light in the near-infrared (NIR) region and generate heat by localised surface plasmon resonance (LSPR) effects and electron–phonon coupling. Optical excitation laser pulses result in hot photocarrier distribution in MXenes, which quickly transfers surplus energy to the crystal lattice and results in the internal conversion of light into heat with nearly 100% efficiency. The relaxation of hot carrier distribution by electron–phonon interactions leads to the cooling of the lattice by dissipating thermal energy to the surrounding environment. This heating effect of MXenes makes them potential photothermal agents (PTAs), particularly for PTT applications. The adjustable surface of MXenes and their high surface area-to-volume ratios are ideal for the combinatorial approach of PTT along with drug delivery, photodynamic therapy (PDT), bone regeneration and other applications. Since non-Ti MXenes are more biocompatible than Ti MXenes, they are promising candidates for different biomedical applications. This comprehensive review provides a concise overview of the current research patterns, properties, and biomedical applications of non-Ti MXenes, particularly in PTT and its combinatorial approaches.

## Abstract 2

This comprehensive review provides a concise overview of the current research patterns, properties, and biomedical applications of non-Ti MXenes, particularly in photothermal therapy and its combinatorial approaches.

## Graphical abstract

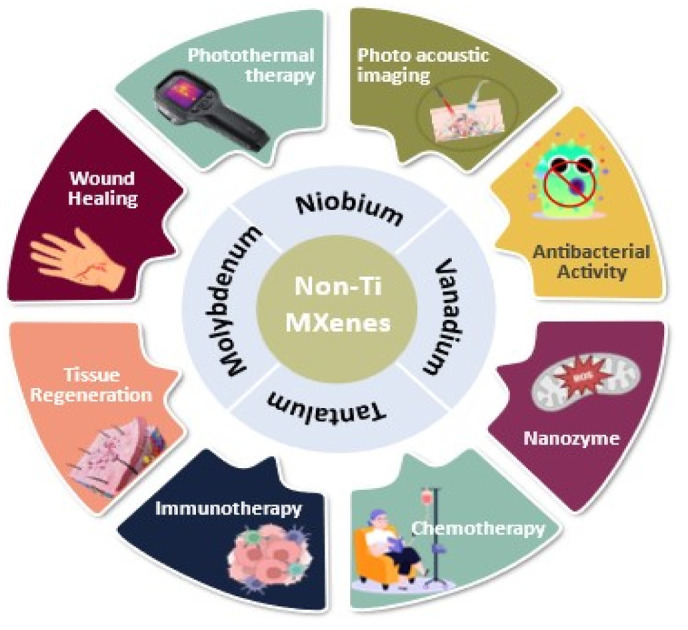

## 1. Introduction

Since the discovery of MXenes in 2011, they have sparked considerable enthusiasm and research interest owing to their adaptable elemental composition, distinctively layered architecture resembling stacked sheets, high surface area and abundant surface terminations offering diverse functionalization possibilities.^1,2^ In comparison with other 2D materials, MXenes have garnered significant attention within the biological field owing to their high biocompatibility, hydrophilicity, light-to-heat conversion performance, mechanical flexibility, *etc.*, sparking a widespread interest in their potential applications in various biomedical fields.^3^ Moreover, MXenes exhibit stronger optical absorption and higher photothermal conversion efficiency (PTCE) than other 2D nanomaterials. As a result, they have emerged as the most promising photothermal agents (PTAs) to achieve effective antitumor therapy.^4^ MXenes have emerged as highly promising materials for various biomedical applications, including photothermal therapy (PTT),^5^ diagnostic imaging,^6^ cancer cell-targeting drug delivery,^7^ and biosensing,^8^ owing to their unique features. Through surface modification, their cytotoxicity can be mitigated, biological stability can be enhanced, and histocompatibility can be improved to enable their potential use *in vivo*. The ability of MXenes to interact with living cells without causing toxicity makes them ideal candidates for drug delivery systems,^9^ tissue engineering,^10^ and biosensing platforms.^11^ The 2D nanoplanar structure of MXenes provides abundant anchor points along with tunable surface functionalities, which makes MXenes excellent candidates for cancer cell-targeting drug delivery.^7^ Furthermore, MXenes demonstrate excellent X-ray attenuation capabilities, making them suitable for applications, such as magnetic resonance imaging (MRI) contrast enhancement and computed tomography (CT) scans.

MXenes are more stable in maintaining the dispersed form than the other 2D materials due to the abundance of surface-terminating hydrophilic groups.^12^ It is easier to design and build MXene-based anticancer nanoplatforms because of their surface functional groups which can provide a large number of reaction sites for the convenient grafting of functional ligands such as nanoparticles, nanoenzymes, and nanosensitizers. These surface functional groups can enhance unique characteristics that are useful in biomedical applications such as drug delivery and therapy.^13^ In addition, the characteristic parameters of nanomaterials such as shape, size, chemical composition, specific surface area, crystal structure, surface morphology, and surface charge can influence the biological interactions and the desired or adverse outcomes of nanomaterial-based delivery and therapy.^14^

With their strong near-infrared (NIR) light absorption and conversion capabilities, MXenes hold promise for tumor ablation *via* PTT, which selectively targets cancer cells while minimizing harm to healthy tissues. MXenes can act as PTAs by the generation of heat *via* localised surface plasmon resonance (LSPR) effects and electron–phonon coupling.^15,16^ Additionally, MXenes can serve as contrast agents for real-time tumor monitoring during cancer treatment.^17^ Modified MXenes can also be engineered to carry and release anticancer drugs in a targeted manner, facilitating synergistic treatments combining PTT and chemotherapy, thereby enhancing the efficacy of cancer treatment significantly.^3^ The biocompatibility and low cytotoxicity of MXenes have been extensively studied, and this is also helpful to design the efficient PTT systems.^18^

## 2. Synthesis of MXenes

**Fig. 1:**
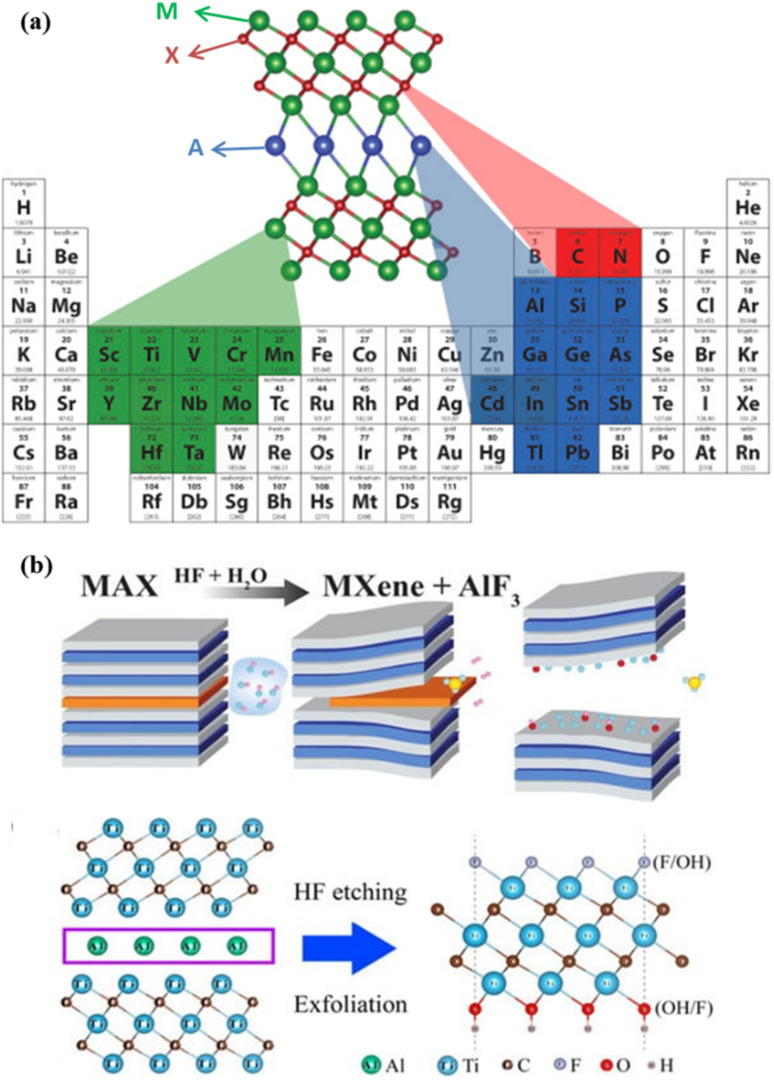
(a) Representation of periodic table with possible M_*n*+1_AX_*n*_ elements. Reprinted with permission from ref. 22. Copyright © 2019 Elsevier Ltd. (b) Schematic of the MXene synthesis by HF etching. Reprinted with permission from ref. 23 Copyright © 2022 by the authors. Licensee MDPI, Basel, Switzerland.

In 2011, Naguib *et al.* synthesized Ti_3_C_2_T_*x*_ MXenes for the first time by the etching of comparatively loosely held aluminium layers from the Ti_3_AlC_2_ MAX phase using strong acid, hydrofluoric acid (HF).^19^ Niobium-carbide MXene is the second representative in the MXene family first discovered in 2013.^20^ Top-down chemical etching is the primary technique employed for synthesizing MXenes, with most precursors used thus far being MAX phases. MXenes are predominantly synthesized through the extraction of A layers from MAX phases, which are ternary carbides or nitrides with the chemical formula M_*n*+1_AX_*n*_, where M is an early transition metal, A indicates a group IIIA or IVA element, X stands for carbon or nitrogen and *n* can be 1–4.^1,21^ About 155 MAX phases have been reported to date. These MAX phases comprise a total of 16 A elements with 14 M elements, as displayed in Fig. 1(a) which shows the possible combinations of M_*n*+1_AX_*n*_ elements.^24,25^ After etching the A layer, MXenes are formed as an array of early transition metal atoms (Ti, Nb, V, Mo, and Ta) stacked with a layer of carbon or nitrogen atoms in between with a formula of M_*n*+1_X_*n*_T_*x*_, where T_*x*_ is a surface functional group such as –O–, –F, –Cl, and –OH, as shown in Fig. 1(b).^26^ The 2D multilayer structure of nanosheets are then made to exfoliate and delaminate further to produce ultrathin or few-layered nanosheets.^27,28^

Nb_2_CT_*x*_ and Nb_4_C_3_T_*x*_ are the most commonly used niobium MXenes, which can be prepared by acid etching from their MAX phases Nb_2_AlC and Nb_4_AlC_3_, respectively.^29^ Other than HF, Xiao *et al.* used an LiF/HCl solution as an etchant for the synthesis of Nb_2_CT_*x*_ MXenes.^30^ The prepared Nb_2_CT_*x*_ MXene exhibited a large interlayer spacing, good surface modification capability and pre-intercalated Li^+^, which can be used for developing high-performance Nb_2_CT_*x*_-based lithium-ion storage devices for the first time. Mo_2_CT_*x*_ MXene nanosheets were synthesized using acids involving the removal of Ga followed by delamination from the Mo_2_Ga_2_C MAX phase.^31^ Mostly Li cations are used to weaken the interlayer attractions between the MXene nanosheets *via* delamination. Organic molecules such as hydrazine, IPA (isopropyl alcohol), *n*-butyl amine, DMF (*N*,*N*-dimethyl formamide), and DMSO (dimethyl sulphoxide) can also be used for the process.^32–34^ MXenes of the type M_4_X_3_T_*x*_ are synthesized mostly *via* HF or LiF + HCl etching.^35,36^ MXenes having two types of metals (Nb_4/5_Ti_1/5_)_4_C_3_T_*x*_ and (Mo_1/2_Ti_1/2_)_4_C_3_T_*x*_ were also reported using HF acid etching.^37,38^ The latest family of MXenes of the type M_5_X_4_T_*x*_ are also etched with HF or *in situ* HF generation.^39,40^

Beyond the standard fluorine-free etching methods, various novel approaches were also investigated for the synthesis of MXenes. In this aspect, J. Mei *et al.* fabricated a fluorine-free Mo_2_C MXene by employing a new UV-induced specific etching technique from the Mo_2_Ga_2_C bulk material.^41^ The obtained MXene possesses a significant level of purity and has great potential for energy storage applications. In another report, Ljubek *et al.* used a mechanochemical treatment using the ball milling technique to acquire Ti_3_C_2_T_*x*_ MXenes from the MAX phase.^42^ Similarly, Y. An *et al.* developed a fluoride- and acid-free physical vacuum distillation method for MXene synthesis.^43^ In this process, a low-boiling-point element was introduced into MAX followed by the evaporation of A elements by physical vacuum distillation. In another report, S. Pang *et al.* synthesized Ti_2_CT_*x*_, Cr_2_CT_*x*_, and V_2_CT_*x*_ with a yield greater than 90% by thermal-assisted electrochemical etching methods.^44^

In a different approach, S. Zada *et al.* employed an environmentally friendly approach to produce V_2_C nanosheets by algae extraction for etching V_2_AlC in an aqueous solution.^45^ The synthesized V_2_C nanosheets (NSs) exhibited excellent structural integrity and displayed exceptional absorption throughout the NIR region, along with a high PTCE. In addition to selective etching and top-down synthesis, alternative bottom-up methods such as chemical vapor deposition (CVD) were also employed. Towards this aspect, D. Wang *et al.* fabricated MXenes using CVD at a temperature of 950 °C on a titanium (Ti) surface using a gas mixture of methane (CH_4_), titanium tetrachloride (TiCl_4_), and argon (Ar).^46^ This directly synthesized MXenes exhibited promising energy storage capacity for lithium-ion intercalation.

Chemical vapor deposition is a viable technique for obtaining high-purity phases of MAX and MXene phases. Xu *et al.* synthesized a nanometer-thick 2D α-Mo_2_C with superconducting properties under ambient conditions by a chemical vapor deposition method.^47^ In another work, Xu *et al.* reported the vertical growth of Mo_2_C on Cu/Mo foil using methane as the carbon source under reducing atmospheres.^48^ Wang *et al.* reported the synthesis of TaB, TaN and TaC using a Cu–Ta support by changing the carrier gas from boron powder, ammonia gas, or ethylene gas respectively.^49^ Owing to the disadvantage of having low yields when synthesized by a CVD technique, template-assisted synthesis provides a viable alternative. Transition metal oxides are usually employed as the template, which is eventually transformed to transition metal carbides or nitrides. Towards this aspect, Joshi *et al.* reported the synthesis of δ-MoN from MoO_3_ nanostructures.^50^

Another category of bottom-up synthetic approaches includes the plasma-enhanced pulsed laser deposition (PEPLD) method. Zhang *et al.* developed highly crystalline and large-crystal Mo_2_C films supported on sapphire as the substrate.^51^ β-Mo_2_C was synthesized by Wolden *et al.* using MoF_6_, hydrogen and oxygen, which were converted into Mo_2_C at 700 °C.^52^ A wide array of MXenes were synthesized *via* molten salt etching in whcih the coupling of molten salt cation occurs with the A element of the MAX phase. Lewis salts such as Ga-, Zn- and Si-based MAX phases were found to be most useful. Thus, new phases of precursors such as V_2_ZnC, Ti_2_ZnC, and Ti_2_ZnN were reported.^53,54^

## 3. Biomedical applications of non-Ti MXenes

**Fig. 2:**
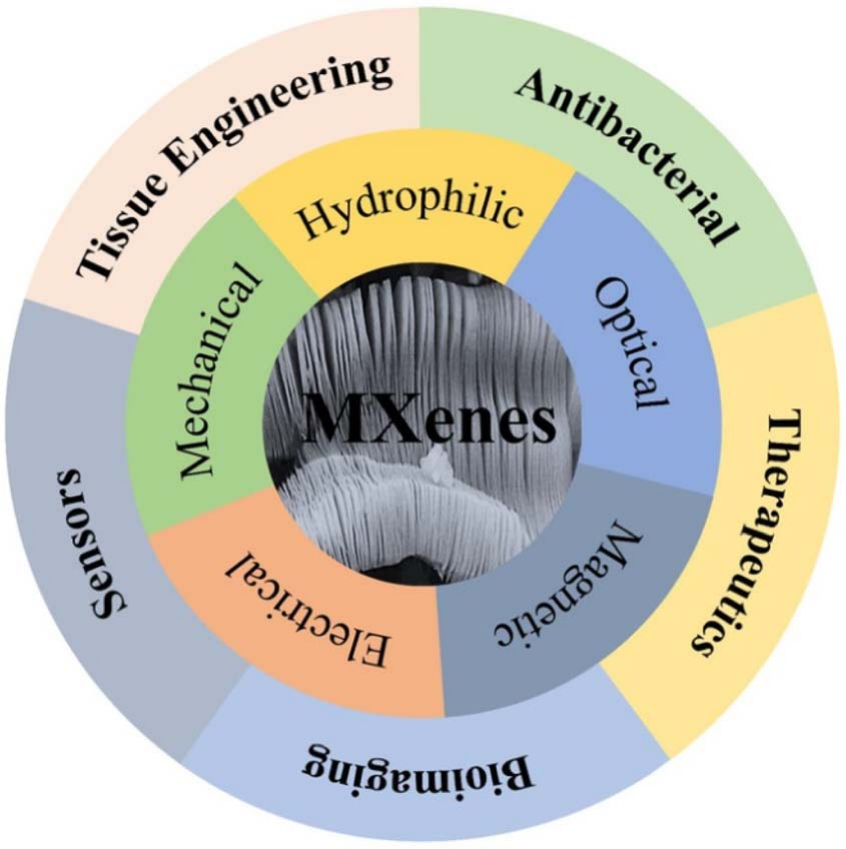
A concise summary of the characteristics and biomedical applications of MXenes. Reprinted with permission from ref. 3 Copyright © 2021 The Authors. Nano Select published by Wiley-VCH GmbH.

In comparison with other 2D materials, MXenes exhibit exceptional electronic properties, outstanding optical properties, promising magnetic properties, superior hydrophilic properties and flexible mechanical properties, which result in various biomedical applications.^3^ Additionally, MXenes possess high biocompatibility, with minimal cytotoxicity and immunogenicity, adaptable surface chemistry and high loading capacity that make them appropriate for various applications in the field of biomedicine including drug delivery, photothermal and sonodynamic therapy and antibacterial applications.^18,55-57^Fig. 2 illustrates the diverse biological applications of MXenes, highlighting their properties that contribute to these applications.

Recently, non-Ti MXenes have emerged as appropriate materials for different applications because of their unique properties such as excellent electrical conductivity, specificity in metallic properties, optical properties such as ultrafast photonic and optoelectronic characteristics, high photothermal stability, and better biocompatibility in comaprison with Ti MXenes. In addition, non-Ti MXenes have better biodegradability, and the physiologically inert transition metal elements in MXenes such as Nb, Mo, V and Ta have previously been shown to have superior biocompatibility.^44,58,59^

For biotechnology applications, niobium-carbide MXenes exhibited better performance with other 2D materials in terms of antibacterial and antifouling activity. It was established that a Nb_2_CT_*x*_-polyethyleneglycol composite does not produce any significant cytotoxicity after incubation with cancer cells even at high concentrations of 300 mg mL^−1^. In addition, the Nb_2_CT_*x*_ MXene showed promising photothermal conversion capability in NIR-I (36.4%) and NIR-II (46.55%) light biowindows in addition to its high photothermal stability and biocompatibility.^60^ Similarly, Nb_2_CT_*x*_-based composites showed a high drug loading efficacy of 32.57% and significant photothermal hyperthermia of cancer with 92.37% inhibition efficiency.^61^

In addition, niobium MXenes showed an ultrafast fast relaxation time (37.43 fs), slow relaxation time (0.5733 ps), and better non-linear optical parameters, and thus, can be used in ultrafast photonic and optoelectronic devices.^62^ Nb_2_CT_*x*_ MXenes exhibited electron–phonon scattering with intensities close to 300 K. This corresponds to phonon–phonon scattering in sharp contrast to a normal case, and this strong electron–phonon scattering can result in a significant reduction in lattice thermal conductivity.^63^

### 3.1. Comparison of non-Ti MXenes with Ti MXenes in terms of biocompatibility

Ti MXenes have mostly been studied for their potential use in biomedical applications. However, there is a lack of understanding about the safety concerns and the impact of Ti MXenes on human health.^64^ As per the study by Wen *et al.*, Ti MXenes can cause abnormal neurobehavior and pathological changes as well as accumulation in organs.^65^ Recently, Wei *et al.* have showed that Ti_3_C_2_T_*x*_ nanosheets disrupt spermatogenesis in mice and high doses of Ti MXenes can cause oxidative stress in the GC-1 cell line.^66^

Non-Ti MXenes with central metal atoms such as Nb, V, and Ta have expected to be shown much better biocompatibility and low cytotoxicity compared to Ti MXenes.^20^ Towards this aspect, L. Fusco *et al.* investigated the effect of Nb_4_C_3_T_*x*_ MXenes and found that Nb_4_C_3_T_*x*_ is not causing any adverse effect on the immune response and the gene expression in the cells.^67^ In another study, H. Lin *et al.* confirmed that the Nb_2_CT_*x*_ nanosheets functionalized with poly vinyl pyrrolidone (PVP) show promising biocompatibility.^60^ The biocompatibility of Nb_2_CT_*x*_ quantum dots (QDs) was further confirmed by Yang *et al.* under *in vitro* and *in vivo* conditions and when incubated with myeloperoxidase and H_2_O_2_.^68^ In addition, Nb_2_CT_*x*_ QDs also showed unique biodegradability responsive to human myeloperoxidase. In another recent study, the toxicity of Nb_2_CT_*x*_ QDs and Ti_3_C_2_T_*x*_ QDs in human umbilical vein endothelial cells (HUVECs) was compared, and it was found that Ti_3_C_2_T_*x*_ QDs result in toxicity towards HUVECs; however, Nb_2_CT_*x*_ QDs displayed no toxicity at the same concentration and incubation time.^69^ The reduced level of IL-6 and IL-8 at the non-cytotoxic level indicates that Nb_2_CT_*x*_ or Ti_3_C_2_T_*x*_ quantum dots did not induce inflammatory responses. It was thus concluded that compared to Ti_3_C_2_T_*x*_ QDs, Nb_2_CT_*x*_ QDs are more biocompatible to HUVECs under the same experimental conditions.

Feng *et al.* found that Mo_2_CT_*x*_ MXene flakes functionalized with poly vinyl alcohol (PVA) display high biocompatibility and biodegradability.^70^ Similarly, the biocompatibility of V_4_C_3_T_*x*_ MXenes was evaluated in immune cells with no cytotoxicity towards the immune cells.^71^ In another work, X. Ren *et al.* have found that PVP-modified Nb_2_CT_*x*_ MXenes can accelerate hematopoietic recovery by enhancing hematopoiesis.^72^ Additionally, metabolic studies showed increased excretion of Nb through feces and urine over time, with no significant toxicity observed in terms of hematology and histology. All these studies point out that non-Ti MXenes are biodegradable and show better biocompatibility compared to Ti-based MXenes.

In addition, Nb_2_CT_*x*_ displays a significant extinction coefficient within the NIR spectrum, aligning with the biologically transparent region. This suggests its potential for utilization in PTT and other biomedical contexts requiring robust interactions with infrared (IR) light. On the contrary, Nb_4_C_3_T_*x*_, Mo_2_Ti_2_C_3_T_*x*_, Ta_4_C_3_T_*x*_, and V_2_CT_*x*_ exhibit comparatively lower extinction coefficients, measuring less than 3000 mL mg^−1^ M^−1^ at 550 nm.^73^ Similarly, the lower thermal conductivity of the Nb_2_CT_*x*_ MXenes in comparison with Ti_3_C_2_T_*x*_, Nb_2_CT_*x*_ and Mo_2_Ti_2_C_3_T_*x*_ has been verified and it can be used in PTT applications with enhanced efficiency.

### 3.2. Photothermal therapy

**Fig. 3:**
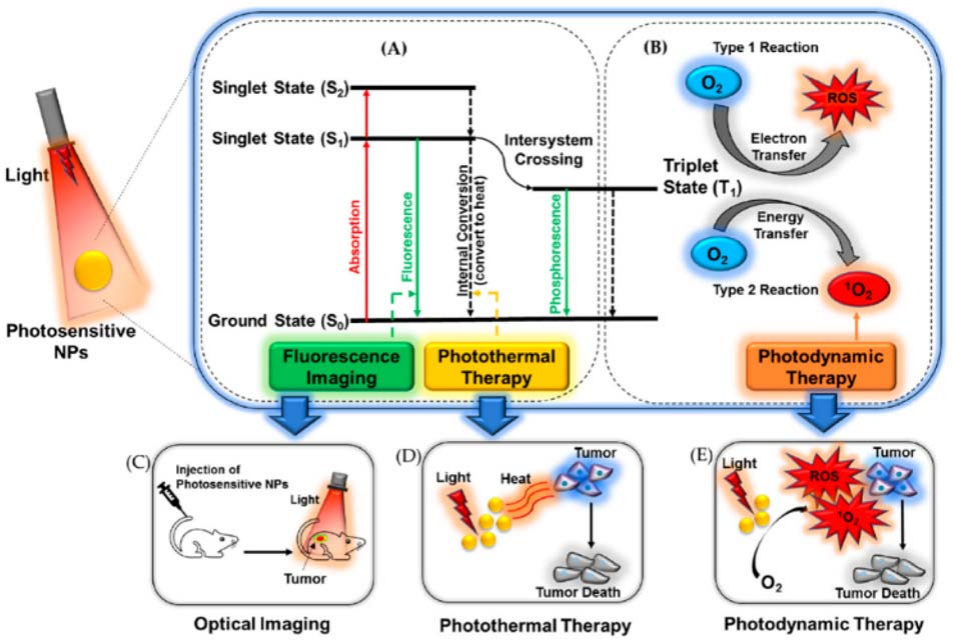
Graphical representation of a figure, which explains the mechanism of optical imaging, photothermal therapy and photodynamic therapy. Reprinted with permission from ref. 75 Copyright © 2020 by the authors. Licensee MDPI, Basel, Switzerland.

When a PTA absorbs a photon, it undergoes electron excitation, which leads to a shift from the ground-state electronic configuration (S_0_) to a singlet excited state. This singlet excited state then rapidly undergoes an internal conversion process to reach the lowest singlet excited state (S_1_).^74^ A PTA at the S_1_ state has the potential to follow three distinct routes for the dissipation of energy, as shown in Fig. 3. The excited electron can undergo nonradiative vibration relaxation to the ground state (S_0_). The relaxation occurs by intramolecular motions and collisions with neighbouring molecules, leading to the production of heat and this process is known as thermal deactivation.^75,76^ The PTT and photoacoustic (PA) imaging primarily rely on the thermal deactivation process to transform light into heat, resulting in localized elevation of temperature. The excited electron can also undergo radiative transition from S_1_ to S_0_, which result in the emission of a photon with a longer wavelength and a lower energy, which is known as fluorescence.^77^ PTA is also susceptible to a transition from the S_1_ state to the lowest triplet state (T_1_) by changing the electron spin multiplicity. This type of transition is referred to as intersystem crossing. At the T_1_ state, molecules have the ability to transition to the S_0_ state by undergoing radiative decay, resulting in the emission of photons. This process is commonly referred to as phosphorescence. Due to its longer lifetime and lower energy level than the S_1_ state, the T_1_ state typically shows delayed and red-shifted emissions, which are characteristic of phosphorescence. The T_1_ state's excited energy has the potential to be transmitted to adjacent oxygen or substrates, resulting in the production of harmful reactive oxygen species (ROS).^78^ These ROS play a crucial role in the photodynamic effect. Since the absorbed excitation energy remains constant for a single molecule, the three dissipation mechanisms are consistently in competition with each other. A graphical representation which explains the mechanism of optical imaging, photothermal therapy and photodynamic therapy is given in Fig. 3.

**Fig. 4:**
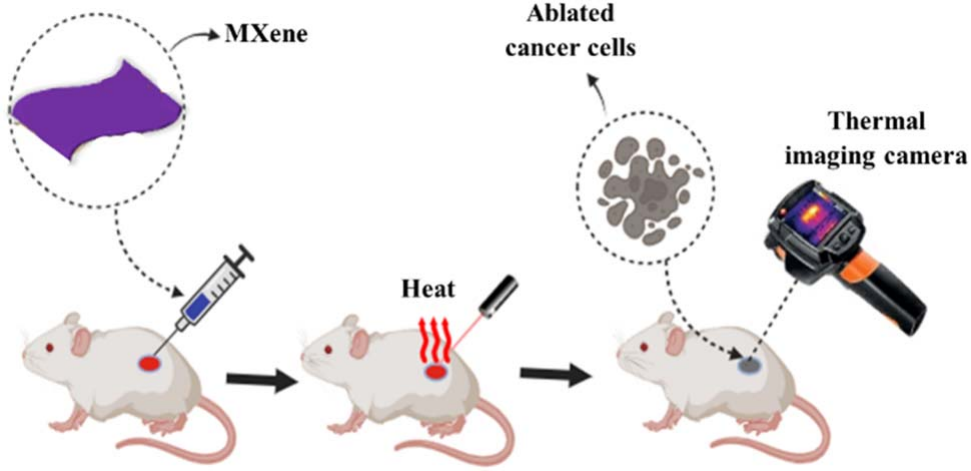
Schematic showing the photothermal ablation of cancer cells using MXenes as PTAs. The authors have drawn the image using https://www.biorender.com/.

The conventional methods of tumor therapy include chemotherapy, radiotherapy, and surgical procedures, which come with numerous disadvantages including severe side effects, limited tumor selectivity, and slow response to treatment. PTT offers the key merits including the capability for deep tissue penetration and high selectivity with minimal effect of surrounding normal cells.^79^ A thermal imaging camera can be used to measure the temperature generated by the PTAs during PTT experiments. As discussed earlier, MXenes emerge as promising PTAs owing to their ability to efficiently absorb energy within the NIR range, subsequently dissipating it as heat.^80^ This unique property enables MXenes to effectively increase the temperature of its surroundings upon exposure to NIR light, making it a valuable tool in PTT applications. The schematics showing the PTT use MXenes as PTAs, which can absorb light energy and convert it into heat energy, as shown in Fig. 4. Apart from cancer treatment, PTT is also used against the bacterial infection. Although antibiotics are used to cure the bacterial infections, bacterial resistance has emerged against the antibiotics, which slow down the efficiency of the treatment.^81^ The PTT can be used as an antibiotic free strategy, which has promising merits of biocompatibility, deep penetration, and highly effective therapeutic efficacy without bacterial resistance.^82^

**Fig. 5:**
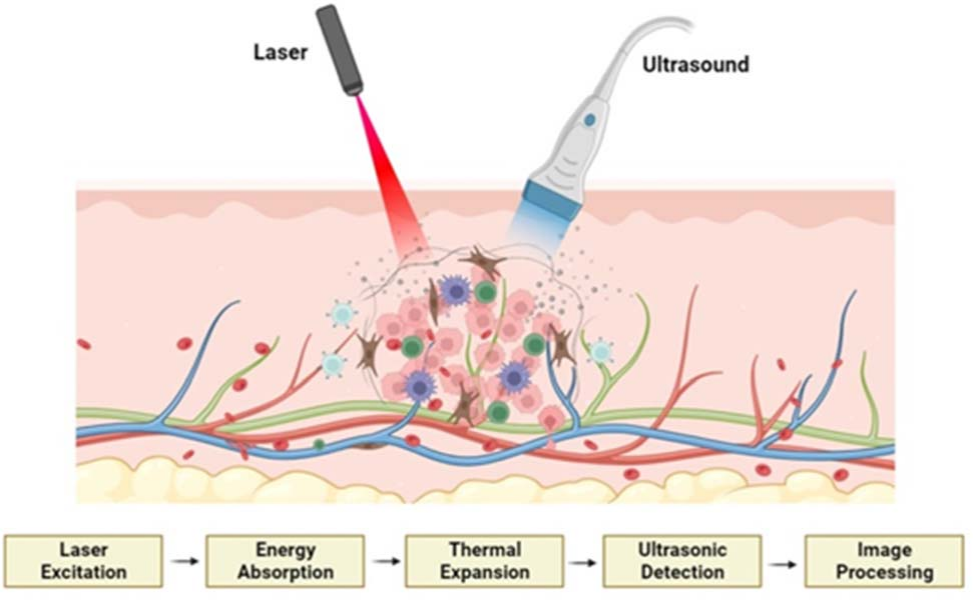
Illustration depicting the process of PA imaging of tumor cells and the underlying mechanism of action. Reprinted with permission from ref. 86 Copyright @ 2022 RSC under CC BY-NC 3.0.

Photoacoustic (PA) imaging is a viable method for measuring the temperature produced during PTT. The first report on the use of photoacoustic imaging for real-time tissue measurement of tissue temperature was published in 2005.^83,84^ PA imaging combines laser light and ultrasound waves to visualize tissues and organs with high resolution.^85^ The change in temperature due to the laser irradiation causes rapid expansion and contraction of the material and the surrounding medium, generating sound waves. This phenomenon allows for imaging based on the optical absorption properties of tissues and materials. Fig. 5 illustrates the mechanism of PA imaging of the tumor cells.

### 3.3. Conditions/properties for MXenes to show photothermal property

The photothermal property evaluation of non-Ti MXenes is still in its infant stage. Towards this aspect, Huang *et al.* confirmed the lower thermal conductivity of Nb_2_CT_*x*_ MXenes, which occurs due to the abnormal strong electron–phonon scattering.^63^ They have used first-principles calculations which show that the Nb_2_CT_*x*_ MXene exhibits strong electron–phonon scattering, which contributes to its high PTCE. In Nb_2_CT_*x*_, the intensity of electron–phonon scattering is comparable to that of phonon–phonon scattering at 300 K, despite the relatively low strength of the electron–phonon coupling. This robust electron–phonon scattering leads to a significant decrease in the lattice thermal conductivity of Nb_2_CT_*x*_. From these reports, it is confirmed that the localized heating originated by strong electron–phonon scattering and Nb_2_CT_*x*_ shows a lower thermal conductivity among the tested MXenes.

In addition, Nb_2_CT_*x*_ displays a significant extinction coefficient within the NIR spectrum, aligning with the biologically transparent region. This suggests its potential for utilization in PTT and other biomedical contexts requiring robust interactions with infrared (IR) light. On the contrary, Nb_4_C_3_T_*x*_, Mo_2_Ti_2_C_3_T_*x*_, Ta_4_C_3_T_*x*_, and V_2_CT_*x*_ exhibit comparatively lower extinction coefficients, measuring less than 3000 mL mg^−1^ M^−1^ at 550 nm.^73^ Similarly, the lower thermal conductivity of Nb_2_CT_*x*_ MXenes in comparison with Ti_3_C_2_T_*x*_, Nb_2_CT_*x*_ and Mo_2_Ti_2_C_3_T_*x*_ has been verified, and it can be used in PTT applications with enhanced efficiency.

## 4. Non-Ti MXenes for PTT and combinatorial therapy approaches

The surface of MXenes usually end with functional groups such as –OH, –O, or –F, depending on the synthesis method.^87^ MXenes have intrinsic hydrophilicity due to these negatively charged terminal groups, which avoids the need for additional modifications. However, the MXenes shows a high degree of susceptibility to oxidation under ambient conditions.^88^ To overcome this barrier, MXenes can be modified with polymer-based molecules such as soybean phospholipid (SP), PVP, polyethylene glycol (PEG), and cellulose hydrogel *via* a physical adsorption reaction over the MXene surface.^89^

Considering the enhanced biocompatibility and biodegradability compared to Ti MXenes, recent research has been focussed on the use of non-Ti MXenes for various biomedical applications including PTT. S. Lin *et al.* introduced a PVP microneedle system loaded with Nb_2_CT_*x*_ and the gradual release of the loaded Nb_2_CT_*x*_ nanosheets occurred after insertion into the skin of the tumor site.^90^ A temperature rise up to 70 °C was observed when irradiated with a 1064 nm laser, and these thermal conditions are sufficient for tumor ablation. The PVP coating provides the promising biocompatibility of the microneedle system and this microneedle system can be used as a minimally invasive, safe and effective strategy for localized superficial cancer treatment.

In conjunction with PTT, other combinations are also being integrated to expand the utilization of MXene for improving the treatment outcomes. Integrating chemotherapeutic drugs with PTT has the potential to enhance cancer therapy by improving the elimination of cancer cells.^61^ MXenes can undergo modifications to enable their utilization in image guidance, ensuring precise accumulation within cancer cells.^6^ In the domain of bone regeneration, experts have investigated the application of MXenes to initiate localized thermal stimulation *in vivo* when exposed to NIR irradiation. Towards this aspect, Yin *et al.* used Nb_2_CT_*x*_ MXenes integrated into the 3D-printed scaffolds that mimic bone structure for the purpose of osteosarcoma treatment.^91^ The photothermal stimulation has demonstrated the ability to elevate the expression of heat shock proteins (HSPs) and facilitate the restoration of bone defects, suggesting promising osteogenic potential.^92^ A temperature greater than 50 °C is sufficient to achieve an efficient tumor-tissue eradication to surpass the thermoresistance induced by HSPs. However, this can also cause damage to nearby normal organs due to the heat diffusion and nonspecific heating.

MXenes exhibit enzyme-like activity by which they can act as peroxidase and oxidase enzymes to catalyse the oxidation of the substrate.^93,94^ Additionally, MXenes serve as a superoxide dismutase and catalase to scavenge excess ROS within cancer cells.^95,96^ When exposed to NIR-II (1064 nm) laser irradiation, this activity reduces hypoxic tumor microenvironment conditions similar to catalase activity, and diminishes glutathione levels as a glutathione-peroxidase analog. This process generates ROS resembling the peroxidase activity, and thus, the catalytic therapy can efficiently inhibit tumor growth.^97^ In addition, the nanozyme activity of MXene to generate ROS that can also be used effectively to eliminate bacteria. Thus, the PTT by using MXene-based systems can enhance both antimicrobial properties and tissue regeneration capabilities.^98^ The following section discusses the use of different non-Ti MXenes such as Nb, Mo, Ta and V MXenes for PTT and combinatorial therapy approaches.

### 4.1. Niobium MXenes

It was observed that Nb_2_CT_*x*_ nanosheets exhibit remarkable PTCE, demonstrating their promising potential for PTT. Using this property, H. Lin *et al.* successfully synthesized Nb_2_CT_*x*_ MXenes with notable photothermal capabilities within both the NIR-I and NIR-II windows.^60^ They achieved a PTCE of 36.4% under the irradiation of 808 nm laser and further demonstrated enhanced performance with a PTCE of 45.65% at 1064 nm along with high photothermal stability. The higher photothermal efficiency observed at the NIR-II window (1064 nm) is attributable to the increased tissue penetration depth in this wavelength range, resulting from reduced absorption and scattering by tissues within the specific NIR-II region. In the *in vivo* experiment, the temperatures at the tumor site exhibited a rapid escalation, reaching approximately 61 °C under 808 nm laser irradiation and around 65 °C under 1064 nm laser irradiation within 10 min duration. The examination of dissected tumor tissues revealed the inhibition of cellular proliferation at various depths of approximately 4 mm. They additionally discovered a pathway for the biodegradation of the synthesized MXene which involves a responsive interaction with the enzyme human myeloperoxidase, leading to the orchestrated degradation of the Nb_2_CT_*x*_ MXene. This enzyme-triggered degradation pathway holds promise for enhancing the biocompatibility and safe clearance of MXene-based materials in biomedical applications.

In another work, a microneedle system incorporating Nb_2_CT_*x*_ and PVP has been synthesized to reduce toxicity within the cancer-affected region by Lin *et al.*^90^ The PVP/Nb_2_C microneedle system on a BALB/c nude mouse resulted in the localized release of Nb_2_CT_*x*_ nanosheets directly into the tumor region by gradual dissolution of the microneedles. The microneedle system, showcasing notable photothermal activity at 1064 nm, has demonstrated the capability to elevate temperatures up to 70 °C upon irradiation with 1 W cm^−2^, highlighting its efficacy in achieving controlled hyperthermic conditions. The cytotoxicity of the raw materials assessed using the standard CCK-8 method indicated that the raw materials exhibited negligible impacts on the growth of 4T1 cells when incubated for one or two days. This observation strongly suggests that the PVP/Nb_2_CT_*x*_ microneedle system is inherently non-toxic, underscoring its biocompatibility for potential biomedical applications.

Another Nb_2_CT_*x*_ composite system has been developed by X. Han *et al.* by introducing a therapeutic mesoporous layer of mesoporous silicon nanoparticles (MSNs) onto the Nb_2_CT_*x*_ sheets with a PTCE of 28.6%.^99^ The introduction of mesopores in the composite facilitated a proficient loading of drugs (32.57%), enhancing its potential for effective drug delivery and targeted therapeutic applications. The mesopore-generating agents, cetanecyltrimethylammonium chloride (CTAC), played dual roles as chemotherapeutic agents, eliminating the need for an extra step of removing these agents and reloading with an alternative drug, thus simplifying the drug-loading procedure. The CTAC@Nb_2_C-MSN composite can rise the temperature to 52.3 °C upon 10 min irradiation of NIR-II region, providing sufficient heat for tumor tissue ablation. The accumulation of the Nb_2_CT_*x*_ nanosheet in the cancer cells was affirmed through PA imaging from *in vitro* and *in vivo* results in which it acts as a contrast agent. This highlights its capability to guide and monitor enhanced therapies, showcasing the promising utility of these MXene composites in biomedical applications. The detection of Nb and Si elements in the faeces and urine of the tested tumor-bearing mice indicates the gradual excretion of these composite nanosheets from the body, which is expected to reduce the risk of accumulation after therapeutic use, ensuring the enhanced biosafety.

Photothermal heating effect of MXenes can also be used to release the drug for developing efficient drug delivery systems. In this regard, X. Lin *et al.* synthesized a Nb_2_CT_*x*_-berberine (BBR) composite by combining the photothermal effect of Nb_2_CT_*x*_ with the chemotherapeutic property of the natural material BBR to eradicate the cancer cells and activate the mitochondrial apoptotic pathway to suppress the cell proliferation.^100^ The heat generated by irradiation of a 1064 nm NIR laser induced the release of BBR, which can modulate the key protein involved in epithelial–mesenchymal transition that is responsible for the lung metathesis. They also discovered that the Nb_2_CT_*x*_-BBR composite primarily entered cells through clathrin-mediated endocytosis to reach subcellular organelles, leading to the suppression of Bcl-2 (an apoptosis inhibitor), elevation of Bax (a pro-apoptotic factor), and reduction in mitochondrial membrane potential. They also utilized the PA imaging properties of Nb_2_CT_*x*_ nanosheets to visualise the localization of the injected Nb_2_C-BBR composite. The standard blood tests, assessments of liver and kidney function, did not indicate any notable toxicity or adverse reactions following two administrations of Nb_2_C-BBR nanocomposites. These findings affirm the favourable biological safety profile of Nb_2_C-BBR nanocomposites while demonstrating their synergistic anti-tumor effects.

The enzyme-like properties of MXenes can be combined with PTT application, which results in enhanced ROS production. In this regard, B. Geng *et al.* synthesized N-doped carbon dots and deposited on the Nb_2_CT_*x*_ sheets with enzyme-like properties to get a photothermal effect at a mild temperature of 43 °C, since a higher temperature may damage the normal cells.^96^ Carbon dots@Nb_2_CT_*x*_ exhibited multifunctional enzyme-like properties, including peroxidase, catalase, and glutathione peroxidase. These capabilities facilitated the generation of a higher amount of ROS to accelerate the termination of cancer cells. The enhancement of the photothermal effect is attributed to the Z-scheme heterojunctions, where electrons are excited from the conduction band of Nb_2_CT_*x*_ to the valence band of the carbon quantum dot nanozyme. The heat generated by the photothermal effects additionally accelerates catalase to produce O_2_, reducing hypoxia in the tumor microenvironment. Simultaneously, peroxidase generates hydroxyl radicals and GSH-Px consumes glutathione (GSH) collectively increasing the generation of ROS. This comprehensive approach of combining the therapy effect of carbon dot@Nb_2_CT_*x*_ nanozymes and mild NIR-II photothermal-enhanced nanocatalytic therapy can contribute to the maximum eradication of the tumor.

**Fig. 6:**
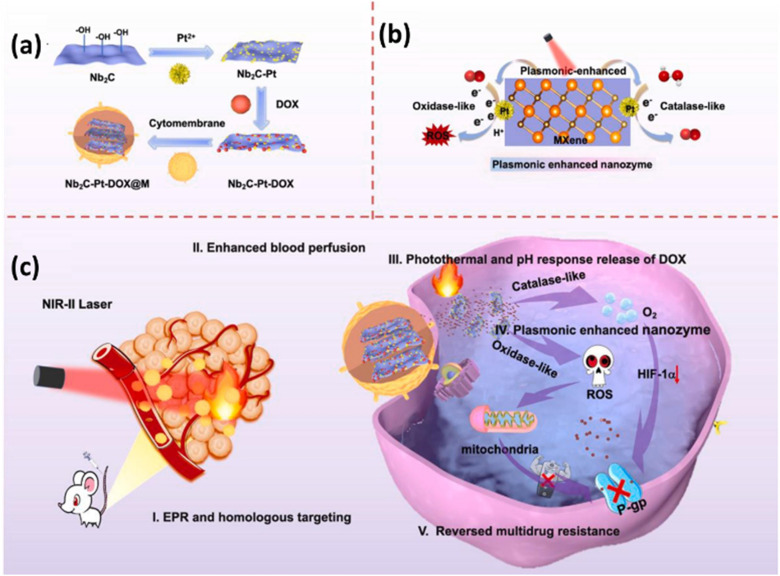
Schematic of (a) development of the biomimetic plasmonic assembly of Nb_2_CT_*x*_, DOX, Pt nanozymes, and tumor cytomembrane, (b) catalytic reaction of plasmonic enhanced nanozyme and (c) graphical abstract of the therapeutic process of plasmonic assembly *in vivo*. Reprinted with permission from ref. 101 Copyright © 2021 Elsevier B.V.

Laser-induced biomimetic plasmonic assemblage based on MXenes has been used for PTT to boost the biocatalytic activity and to achieve complete tumor destruction. Hao *et al.* introduced an assembly of Nb_2_CT_*x*_ plasmon, doxorubicin (DOX), Pt nanozymes, and tumor cytomembrane for targeted cancer treatment.^101^ Here, the NIR laser exposure facilitates the catalase-like and oxidase-like functions of Pt nanozymes to generate ROS, which are crucial for eradicating deep-seated tumor cells. The Pt nanoenzyme increases oxygen production in response to the elevated H_2_O_2_ levels within the tumor site and effectively lowers the condition of tumor hypoxia and modulates the production of hypoxia-inducible factor (HIF-1α). The increased oxidase activity contributes to enhanced ROS production, potentially disrupting mitochondrial energy supply to P-glycoprotein, a vital membrane efflux pump responsible for recognizing and expelling chemotherapeutic drugs from cells. Furthermore, DOX is released in response to the acidic conditions prevailing within the tumor microenvironment. The overview of the development of biomimetic plasmonic assembly, catalytic process of plasmonic enhanced nanozyme, and the therapeutic process of the plasmonic assembly *in vivo* is given in Fig. 6. This comprehensive approach underscores the potential of the Nb_2_CT_*x*_ plasmonic assembly in combatting cancer through a network of interconnected mechanisms.

By using the polydopamine (PDA)-coated Nb_2_CT_*x*_ sheets along with immunotherapeutic drug (R837), Y. Lu *et al.* improved the effect of cancer therapy through the synergistic combination of immunotherapy and PTT.^102^ The use of the 1064 nm laser conferred a dual advantage, as it not only increased the temperature of cancer cells but also facilitated the burst release of R837. They accomplished a PTCE of 27.6% and attained a temperature of 57 °C within 5 min. The PDA-coated Nb_2_CT_*x*_ sheets loaded with R837 were subsequently enveloped in a red blood cell (RBC) membrane, effectively preventing the coagulation of the Nb_2_CT_*x*_ sheets and, thereby, prolonging the blood circulation. The incorporation of the RBC membrane further facilitated a higher cellular uptake, leading to an increased level of cellular apoptosis. The drug R837 initiated the release of tumor-associated antigens, promoting the maturation of dendritic cells, and inhibited the primary tumor growth.

PTT faces the challenge of incomplete accumulation of the PTAs in cancer cells, leading to a reduction in efficiency. B. Zhou *et al.* endeavoured to overcome this challenge by synthesizing a composite that incorporates the Nb_2_CT_*x*_ MXene as a PTA, along with a phase-changing plant-origin protein, zein.^103^ Upon exposure to water at the tumor site, the ethanol-dissolved zein undergoes a phase transition to a solid state. This transition entraps the Nb_2_CT_*x*_ MXene to ensure complete localization of Nb_2_CT_*x*_ within the tumor site, thereby enhancing the efficiency of PTT. The temperature increases to 65 °C within 5 min upon irradiation with a 1064 nm NIR laser. Cancer cells are additionally eradicated when the solidified zein obstructs the blood vessels in the tumor site, thereby preventing the blood supply to the tumor and inducing tumor starvation.

The efficiency of cancer therapy is significantly heightened when the synthesized therapeutic material not only eradicates cancer cells but also contributes to the regeneration of damaged cells. In this approach, J. Yin *et al.* developed a versatile framework incorporating Nb_2_CT_*x*_ sheets into a 3D bioactive glass scaffold with a specific focus on the photonic induction of bone tumor hyperthermia and enhanced bone regeneration.^104^ The temperature increased to 56 °C within 3 min of irradiation with a 1064 nm laser, resulting in a 62% cellular inhibition. They utilized the biodegradable characteristics of the 3D bioactive glass scaffold to enable the controlled release of calcium and phosphate, facilitating the bone regeneration process. When the calcium phosphate ratio in the scaffold closely resembles hydroxyapatite, superior performance in bone regeneration is achieved, attributed to enhanced osteogenic differentiation and bone mineralization. The degradation of the scaffold led to the release of Nb_2_CT_*x*_ MXenes, which facilitated the formation and movement of blood vessels at the location of injury. This enhancement in vascularity could facilitate the transport of oxygen and vitamins around the bone defect, thereby amplifying the regenerative processes. Hence, this engineered multifunctional scaffold offers a unique biomaterial scaffold that showcases concurrent capabilities in tumor therapy and the regeneration of bone tissue.

Beyond the direct induction of osteogenesis through heat, Q. Yang *et al.* demonstrated that the gas released during NIR-II radiation can effectively stimulate bone growth by facilitating the development of blood vessels within newly formed bone tissues.^105^ They integrated a mesoporous silica-coated Nb_2_CT_*x*_ MXene loaded with *S*-nitrosothiol (a nitric oxide donor) into porous 3D-printed scaffolds for the dual purpose of bone regeneration and osteosarcoma eradication. Here, the NIR-II hyperthermia not only directly eliminated tumor cells but also triggered the explosive release of nitric oxide (NO) from *S*-nitrosothiol, primarily inducing DNA damage through oxidative and nitrosative stress for effective tumor cell destruction. The low concentration of NO following PTT played a pivotal role in modulating mesenchymal stem cells and endothelial cells within 3D-printed scaffolds, promoting angiogenesis and fostering bone regeneration.

The bone metastasis of breast cancer poses a significant clinical challenge, and existing treatments are notably destructive. In order to eliminate primary/metastases tumors and enhance bone–tissue regeneration, He *et al.* constructed an immune adjuvant (R837)-loaded Nb_2_CT_*x*_-modified 3D-printing biodegradable scaffold.^106^ The immune adjuvant (R837) was loaded into the mesopores formed by the silica layer on Nb_2_CT_*x*_ sheets, and subsequently integrated into a biodegradable bio-glass scaffold. They found that tumors were destroyed by irradiation with an 808 nm laser, and the immune adjuvant R837, along with tumor debris released during thermal ablation, facilitated the maturation of dendritic cells and stimulated cytokine secretion. This activation triggered an immune response to target tumors by establishing long-term protection to avoid tumor reoccurrence. The scaffold composite showcased a significant osteogenic potential, attributed to their three-dimensional structure and thus accelerated osteogenesis followed by the removal of tumors.

**Fig. 7:**
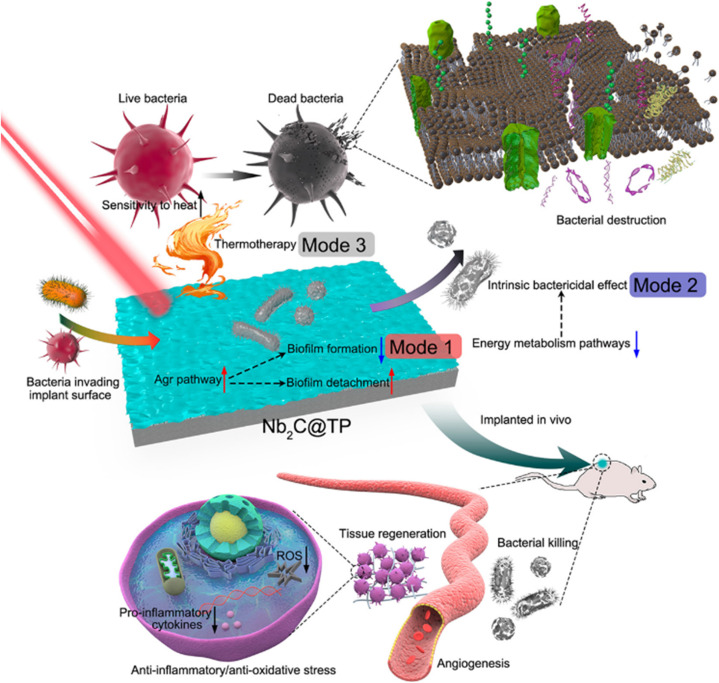
Diagram of the trimodal bacterial killing strategy of Nb_2_CT_*x*_@TP and their *in vivo* tissue regeneration properties. Reproduced with permission from ref. 98. Copyright © 2021, the American Chemical Society.

Photothermal therapy employing Nb_2_CT_*x*_ MXenes not only holds promise for cancer treatment but also showcases effectiveness in eliminating bacterial infections. Towards this aspect, C. Yang *et al.* designed Nb_2_CT_*x*_ MXene nanosheets in combination with titanium plates (TPs), facilitating antimicrobial eradication and tissue regeneration capacities.^98^ The temperature of the synthesized system can elevate to 55 °C within 2 min under the irradiation of an 808 nm laser and effectively eradicate planktonic bacteria by impairing their motility systems. This thermal therapy not only causes bacterial death *in vivo* but also moderates severe inflammatory responses and reduces ROS production, thereby promoting angiogenesis and restoration of damaged tissues. However, achieving the complete eradication of the biofilm solely *via* heat is not feasible. Consequently, they proposed a trimodal antibacterial strategy that integrates biofilm prevention with inherent bactericidal effects and photothermal bacteria ablation. Nb_2_CT_*x*_@TP activates the gene regulator (Agr) when bacteria attempt to invade the implant surface, and thus, it hinders the bacterial attachment and facilitates the removal of pre-existing biofilms. Additionally, Nb_2_CT_*x*_@TP directly induces bacterial death by downregulating key metabolism pathways, including the tricarboxylic acid (TCA) cycle and phosphotransferase system (PTS) pathway. This multifaceted approach, encompassing antimicrobial eradication, tissue regeneration, and a trimodal antibacterial strategy, underscores its potential significance in advancing infection control methodologies, as shown in Fig. 7.

In another work, H. Yuan *et al.* achieved a highly efficient antibacterial activity by combining the photothermal properties with the enzyme catalytic property.^107^ They synthesized the CeO_2_/Nb_2_CT_*x*_ nanocomposite to utilize the photothermal property of Nb MXenes and peroxidase-like nanozyme activity of CeO_2_. The CeO_2_/Nb_2_CT_*x*_ nanocomposite exhibited a PTCE of 38%, and the synergistic effect of PTT and chemical dynamic therapy (CDT) maximized the antibacterial efficacy. The ROS generated by PTT were harnessed by CDT, which amplified hyperthermia and enhanced bacterial destruction. In addition to their peroxidase-like property, CeO_2_ nanoparticles exhibit antioxidant activity which promotes cell proliferation within wounds and expedites the closure of wounds by shielding cells from excessive ROS during the later stages of the wound-healing activity. The summary of Nb MXene-based PTAs in treating cancer by PTT alone and their combinatorial therapy are given in Table 1.

**Table 1:** Summary of Nb MXene-based PTAs in treating cancer by PTT alone and their combinatorial therapy

PTA	Therapeutic modality	Ref.
PVP microneedle system with Nb_2_CT_*x*_ nanosheet	PTT alone	90
Nb_2_CT_*x*_ nanosheet in conjugation with PVP	PTT at NIR-I and NIR-II window	60
Nb_2_CT_*x*_ MXene with *S*-nitrosothiol (RSNO)-grafted mesoporous silica with 3D-printing bioactive glass (BG) scaffolds	PTT for bone cancer and bone regeneration	105
Mesopore coated Nb_2_CT_*x*_ MXene	PTT and chemotherapy	99
Phase changeable bio injection with Nb_2_CT_*x*_ MXene with plant protein zein	PTT and shear wave elastography (SWE) (deep-penetration imaging mode)	103
CeO_2_/Nb_2_CT_*x*_nanocomposite	PTT for diabetic wound bacterial infection	107
N-doped carbon dots onto Nb_2_CT_*x*_ nanosheets	PTT with triple enzyme–mimic activities to obtain amplified ROS levels	96
Nb_2_CT_*x*_ MXene on titanium plate	PTT for bacterial infection elimination and tissue regeneration	98
Nb_2_C@PDA-R837@RBC nanoparticles	PTT and immune therapy	102
Nb_2_CT_*x*_ MXene nanosheets (NSs) into the 3D-printed bone-mimetic scaffolds (NBGS)	PTT for osteogenesis, osteoconduction and osteoinduction, and bone regeneration	91
R837 (immune adjuvant) loaded on Nb_2_C@Si nanosheet on 3D printed bioglass	Bone metastasis of breast cancer	106
Plasmonic assembly of Nb_2_CT_*x*_ plasmon (MXene), Pt nanozymes, DOX and tumor cytomembrane	PTT and chemotherapy	101

J. Chin *et al.* developed an injectable thermosensitive hydrogel (Nb_2_C@Gel) using Nb_2_CT_*x*_ and a poly(lactic-*co*-glycolic) acid (PLGA)-PEG-PLGA triblock copolymer for diabetic wound healing.^108^ The Nb_2_CT_*x*_@Gel system exhibits excellent PTCE and *in vivo* as well as *in vitro* antimicrobial ability. When the infected wounds treated with Nb_2_CT_*x*_@Gel are exposed to NIR laser irradiation, the temperature at the wound site increases significantly, indicating the effective photothermal property of Nb_2_CT_*x*_@Gel. This increase in temperature effectively eliminates bacteria in the infected wounds, as demonstrated by the significant reduction in bacterial survival rate after NIR laser irradiation. Furthermore, the photothermal effects of Nb_2_CT_*x*_@Gel help in alleviating the inflammatory response and oxidative stress induced by infection, hence promoting angiogenesis and tissue regeneration. Additionally, the over activation of inflammatory responses, which leading to excessive formation of ROS, is reduced by the photothermal property of Nb_2_CT_*x*_@Gel. This property enables the scavenging of excess ROS produced due to infection, thus reducing the damage caused by oxidative stress and inhibiting an inflammatory response.

### 4.2. Vanadium MXenes

Recently, V_2_CT_*x*_ MXenes have also emerged as promising candidates for PTT and other applications.^109^ In this approach, S. Wu *et al.* designed a V_2_CT_*x*_-DOX nanocomposite for the targeted eradication of triple-negative breast cancer.^110^ The temperature of the cancer cells increased to 55 °C within 5 min of irradiation of 808 nm laser. The photothermal effect not only elevated the temperature of the cancer cells but also facilitated the enhanced release of the chemotherapeutic drug, DOX, which further kills the cancer cells. They also observed that the treatment involving V_2_CT_*x*_-DOX and laser may induce significant changes in gene expression, and thus, contribute to the demonstrated anti-breast cancer activity. In another work, low-temperature (45 °C) PTT was realized by combining V_2_CT_*x*_ QDs with an engineered exosome (Ex) vector for nucleus-targeted low-temperature PTT.^111^ The QDs engineered for the dual-target cancer cell membrane and nucleus were modified with the nucleus-targeted TAT peptide and the cell-targeted RGD peptide. The V_2_CT_*x*_-TAT@Ex-RGD composite utilized the endocytic uptake pathway to interact with the target cancer cells. The cellular uptake of the composite was explored by employing its photoluminescence property, which results from the edge effect and quantum confinement. Owing to the quantum mechanical confinement and 3d^1^ electronic configuration of vanadium, the V_2_CT_*x*_-TAT@Ex-RGD system could also serve as a contrast agent for MRI. Here, the efficient tumor accumulation of V_2_CT_*x*_-TAT@Ex-RGD was accomplished through its extended blood circulation time and potent cancer cell targeting ability, resulting in compelling antitumor therapeutic efficiency.

To avoid the use of toxic HF as an etching agent, S. Zada *et al.* employed algae extraction as a delamination agent to produce V_2_CT_*x*_ nanosheets, ensuring strong structural integrity and enhanced PTT activity.^45^ The V–Al bonds were effectively disrupted by the organic acids present in the algae extraction mixture (pH = 2.3), which, in turn, resulted in the release of bioactive compounds, thereby further accelerating the delamination process. The synthesized V_2_CT_*x*_ sheets showed a notable PTCE of 48%, resulting in a temperature elevation of cancer cells to 57.9 °C after 10 min of exposure to an 808 nm laser. The MRI and PA signals exhibited by the V_2_CT_*x*_ nanosheets at lower concentrations indicate their excellent imaging capabilities for tumor diagnosis. The outcomes of cytotoxicity analysis, hemolysis assay, and the Calcein AM/PI double staining method suggest the excellent biocompatibility of the V_2_CT_*x*_ nanosheets. The same research group evaluated the antibacterial activity of the V_2_CT_*x*_ nanosheets synthesized by using algae extracts and evaluated their high antibacterial efficiency through PTT.^112^ Following the injection of 40 μg mL^−1^ of V_2_CT_*x*_ NSs, exposure to an 808 nm laser increased the temperature to 52.34 °C, leading to the confinement of bacteria within V_2_CT_*x*_ NSs. The cell walls were stripped down, and the release of cytoplasmic materials from cells was observed through the lactate dehydrogenase (LDH) release assay. A low-dose V_2_CT_*x*_ NS suspension (40 μg mL^−1^) coupled with 5 min of NIR irradiation successfully eliminated around 99.5% of both bacterial species, attributed to the inherent antibacterial properties and photothermal effects of V_2_CT_*x*_ NSs.

**Fig. 8:**
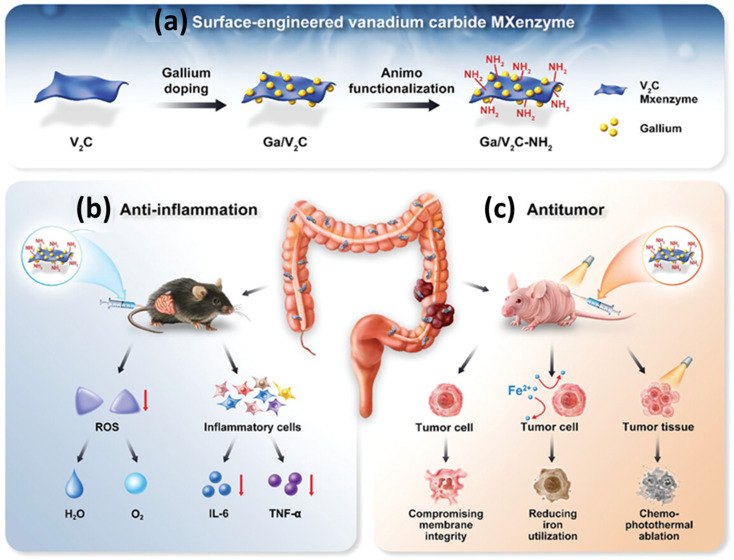
(a) Schematic of the development of surface-engineered V_2_CT_*x*_ MXenzymes, (b) their inherent ability to scavenge ROS and exhibition of photothermal activities for anti-inflammation and (c) antitumor treatment in colon diseases. Reprinted with permission from ref. 113 Copyright © 2023 Wiley-VCH GmbH.

A certain type of cancer is associated with inflammation-induced pathways, and thus, chronic inflammation can induce the cancer progression. To effectively address the issues with inflammation, J. Deng *et al.* have engineered V_2_CT_*x*_ MXenzymes with gallium doping and amino functionalization for efficient anticancer therapy.^113^ The introduction of amino groups to functionalize V_2_CT_*x*_ imparts a positive charge, facilitating its targeted binding to negatively charged tumor cells. Gallium demonstrates a significant potential as an anticancer agent by substituting iron within bacteria and disrupting their function. By utilizing the multiple enzyme-mimicking activities, V_2_CT_*x*_ MXenzymes are able to reduce excessive ROS and inhibit the levels of proinflammatory cytokines, IL-6 and TNF-α produced, thereby promoting the decomposition of ROS which resulted in reduced proinflammatory cytokine levels. The surface-engineered Ga/V_2_CT_*x*_-NH_2_ composite also exhibits photothermal properties with the temperature increased to 56.3 °C within 10 min of irradiation with an 808 nm NIR laser. This irradiation also led to a greater release of gallium, and thus, enhanced the efficiency of chemophototherapy. The schematic depiction of the development of surface-engineered V_2_C-MXenzymes and their inherent abilities to scavenge ROS and exhibit photothermal activities for anti-inflammation and antitumor treatment is given in Fig. 8. The injected Ga/V_2_CT_*x*_-NH_2_ in the mouse model disappeared from all the representative organs after 48 h, thereby underscoring its complete *in vivo* clearance and elucidating its exceptional biosafety. The stability of Ga/V_2_CT_*x*_-NH_2_ nanosheets persisted for a minimum of 1 month when it is preserved at 4 °C under N_2_ protection, maintaining their photothermal ability and unaltered enzyme-like activity.

In another work, a multifunctional nanoplatform made of V_4_C_3_T_*x*_/atovaquone@bovine serum albumin (V_4_C_3_T_*x*_/ATO@BSA) increased nanozyme catalytic/photothermal therapy to disturb the tumor redox homeostasis for amplified nanozyme PTT.^114^ V_4_C_3_T_*x*_ as a PTA and atovaquone (ATO) as HSP suppressor are encapsulated within the biocompatible BSA. Owing to the photothermal effect and the mixed state of vanadium in V_4_C_3_T_*x*_, vanadium-bonded ATO exhibits enhanced peroxidase-like activity to generate ROS. Additionally, the presence of vanadium states of composites contributes to the consumption of GSH, thereby increase the ROS concentration within the tumor microenvironment. The elevated expression of GSH in the tumor microenvironment triggers the cleavage of the disulfide bond in BSA, leading to the release of V_4_C_3_T_*x*_ and ATO. The ATO binds to mitochondrial cytochrome reductase, suppresses the ATP synthesis, and consequently downregulates the expression of HSPs, and this results in a reduction of heat resistance and enhances the efficiency of PTT. The PA imaging properties of the V_4_C_3_T_*x*_-based system can be used to evaluate the therapeutic process.

W. Zhao *et al.* investigated the approaches to minimize exposure duration and achieve effective sterilization by using V_2_CT_*x*_ nanosheets.^115^ The V_2_CT_*x*_ solution reaches a temperature of 72.5 °C with a laser power of 1.5 W within 8 min. The V_2_CT_*x*_ solution exhibits higher sensitivity to Gram-positive *Staphylococcus aureus* (*S. aureus*) compared to Gram-negative *Escherichia coli* (*E*. *Coli*) owing to the thicker peptidoglycan film of *S. aureus*, which has a sticky property, which allows for increased contact with V_2_CT_*x*_, resulting in more efficient eradication. The presence of lipopolysaccharide in the outer membrane of *E. coli* may induce steric repulsion, weakening the contact with the V_2_CT_*x*_ material and potentially impacting its antibacterial effectiveness. In this work, effective photothermal sterilization, with a PTCE of 45.15%, can be achieved using a lower dose of 20 μg mL^−1^ of V_2_CT_*x*_.

The photothermal effects against biofilm bacteria have been improved through the synergistic interplay of membranolytic and photocatalytic activities facilitated by a V_2_CT_*x*_ MXene-based microneedle system by Feng *et al.*^116^ They engineered a microneedle array incorporating a four-armed poly(l-lysine) (4K10)-enhanced V_2_CT_*x*_ MXene (4K10@V_2_CT_*x*_ MXene) to eliminate multidrug-resistant (MDR) bacterial infections. The electrostatic repulsion between the negatively charged V_2_CT_*x*_ MXene and bacteria is mitigated by coating the MXene with positively charged poly(l-lysine). This coating improves the accessibility of bacterial cell membranes, and thus, enhances the effectiveness of PTT. The presence of 4K10 and the photothermal effects of V_2_CT_*x*_ demonstrated robust antibacterial activity by disrupting the bacterial membranes, which resulted in direct interaction between bacterial cells and antimicrobials. Upon UV-visible light irradiation, V_2_CT_*x*_ MXene nanosheets can undergo oxidation and degradation and transform into bioactive V_*x*_O_*y*_. This process leads to the persistent generation of ROS, which helps to inhibit the reoccurrence of bacterial growth. The biodegradability of 4K10 and V_2_CT_*x*_ ensure that there will be no prolonged accumulation of nanocomposite systems.

Besides vanadium carbide MXenes, vanadium nitride MXenes have been used as PTAs for PTT applications. X. Sun synthesized a V_2_NT_*x*_ MXene, which functions both as a PTA and as a nanozyme, exhibiting superoxide and peroxidase-like behaviour.^97^ The temperature increased up to 47.8 °C under the irradiation with 1064 nm with a PTCE of 31.67%. The photothermal effect further amplifies the nanozyme catalytic activity of V_2_NT_*x*_, leading to the generation of ROS to effectively destroy the bacteria. The summary of vanadium MXene-based PTAs in treating cancer by PTT alone and their combinatorial therapy is given in Table 2.

**Table 2:** Summary of vanadium MXene-based PTAs in treating cancer by PTT alone and their combinatorial therapy

PTA	Therapeutic modality	Reference
V_2_NT_*x*_ MXene	Photothermal-enhanced dual enzyme-like activities for anti-infective therapy	97
V_2_CT_*x*_ –DOX nanocomposite	PTT and chemotherapy for triple negative breast cancer	110
Vanadium carbide quantum dots (V_2_CT_*x*_ QDs) PTA combined with engineered exosomes (Ex) vector	Low-temperature nucleus-targeted PTT	111
V_2_CT_*x*_ MXene	PA imaging and MRI guided PTT	45
V_2_CT_*x*_ nanosheets	Antibacterial activity through PTT	112
V_2_CT_*x*_ nanoenzyme with amino functionalization and gallium doping (Ga/V_2_CT_*x*_-NH_2_)	Anti-inflammation and photoenhanced antitumor therapy of colon diseases	113
V_4_C_3_T_*x*_/atovaquone@bovine albumin	Catalytic PTT	114
V_2_CT_*x*_ MXene	PTT of Gram-negative and Gram-positive bacteria	115
Four-armed host-defense peptidomimetic 4 K10 on V_2_CT_*x*_ MXene	Membranolytic-photothermal effects against biofilm bacteria	116
Mo_4_VC_4_T_*x*_ MXene	PTT-CDT synergistic strategy for MRSA and *Pseudomonas aeruginosa* bacteria	117

A new type of MXene with M_5_C_4_ phases (Mo_4_VC_4_T_*x*_) was synthesized to utilize the synergistic functions of PTT and CDT against *Pseudomonas aeruginosa* (*P*. *aeruginosa*) as well as methicillin-resistant *S. aureus* (MRSA).^117^ The Mo_4_VC_4_T_*x*_ MXene exhibits hyperthermia by NIR-II treatment by PTT and produce highly efficient ˙OH radicals by CDT, which can kill bacteria efficiently. An antibacterial rate of 99.8% for *P. aeruginosa* and 99.5% for MRSA in the infected wound was achieved and this significantly accelerated the wound healing.

### 4.3. Tantalum MXenes

**Fig. 9:**
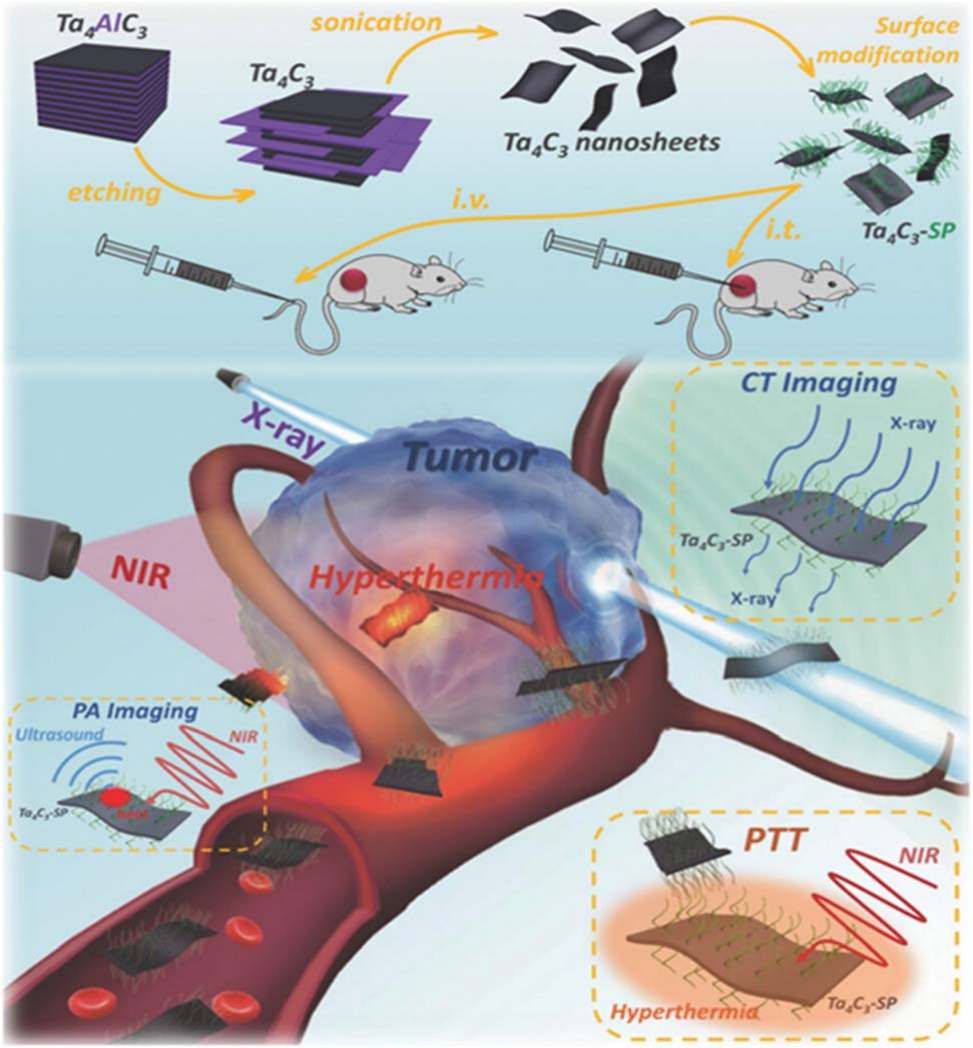
Schematic of the synthesis of Ta_4_C_3_T_*x*_ nanosheets by delamination, surface SP manipulation, and *in vivo* PA/CT dual-mode imaging combined with PTT. Reprinted with permission from ref. 119 Copyright © 2017 WILEY-VCH Verlag GmbH & Co.

Tantalum Carbide (Ta_4_C_3_T_*x*_) nanosheets exhibit strong NIR absorption and deliver improved contrast for CT imaging due to a high atomic number *Z* = 73, as compared to other MXenes.^118^ Furthermore, Ta_4_C_3_T_*x*_ MXenes also exhibit significantly comparable PTCE with Nb_2_CT_*x*_ and Ti_3_C_2_T_*x*_ MXenes. Lin *et al.* studied the dual-mode PA/CT imaging using Ta_4_C_3_T_*x*_ MXenes and their incredible efficacy *in vivo* photothermal ablation of mouse tumor xenografts.^119^ They have showed that SP-modified Ta_4_C_3_T_*x*_ nanosheets are able to demonstrate NIR photothermal performance with an exceptionally high PTCE of 44.7%, with promising photothermal stability. The Ta_4_C_3_T_*x*_-SP nanosheets possess a significant ability to absorb X-rays and NIR light, making them a promising contrast agent for tumor imaging using CT scans. The schematic illustration showing the synthesis and SP modification of Ta_4_C_3_T_*x*_ nanosheets as well as the combination of PTT along with *in vivo* PA/CT dual-mode imaging is given in Fig. 9.

In a different work, Liu *et al.* altered the Ta_4_C_3_T_*x*_ surface by growing superparamagnetic Fe_3_O_4_ NPs (IONP) *in situ* and then coated it with SP, which demonstrated improved results in PTT in the NIR region.^118^ They found that Ta_4_C_3_-IONP-SP composite showed high PTCE (32.5%) to achieve entire elimination of tumors without any recurrence. The Ta component of Ta_4_C_3_T_*x*_-IONP-SP provides high performance for enhancing contrast in CT imaging and the presence of superparamagnetic IONPs helps it to act as an excellent contrast agent for MR imaging. In another work, manganese oxide nanoparticle (MnO_*x*_)-Ta_4_C_3_T_*x*_ nanocomposite (MnO_*x*_/Ta_4_C_3_T_*x*_) has been used for several imaging-guided photothermal tumor ablation.^120^ The MnO_*x*_/Ta_4_C_3_T_*x*_-SP nanosheets exhibited a higher PTCE of 34.9%, which indicates their excellent photothermal-hyperthermia efficacy for tumor ablation. The Ta components of the composite acted as the high-performance contrast agents for CT imaging and the integrated MnO_*x*_ component acted as contrast agents for MR imaging.

In contrast to tantalum carbide MXenes, tantalum nitride-based MXenes (TaNT_*x*_) exhibit remarkable PA contrast effect and photothermal characteristics, indicating its potential for use in combination of therapy and theranostic applications. Pu Lin *et al.* developed TaNT_*x*_ nanosheets as theragnostic agents for photothermal tumour elimination guided by PA imaging.^121^ PEG was used to functionalize the TaNT_*x*_ nanosheets and the TaNT_*x*_-PEG nanosheets demonstrate excellent biocompatibility and stability in biological systems. The experimental results confirmed that TaNT_*x*_-PEG is capable of producing substantial enhancement of photoacoustic contrast due to its broad absorption in the NIR region.

### 4.4. Molybdenum MXenes

Mo_2_CT_*x*_ MXenes are increasingly recognized as a viable option for advancing PTT in current research investigations and their unique combination of high PTCE and excellent biocompatibility make them ideal for targeted cancer treatment. Q. Zhang *et al.* developed Mo_2_CT_*x*_ nanospheres that exhibit dual functionality upon NIR radiation, as they generate heat and also have the ability to produce ROS.^122^ This makes the synthesized material serve as both a PTA and a photosensitizer. The singlet oxygen quantum yield of Mo_2_CT_*x*_ at 1064 nm was measured to be 0.0044, surpassing that of the widely recognized photosensitizer indocyanine green (ICG). The combination of PDT/PTT using NIR-triggered Mo_2_CT_*x*_ led to an 80% cancer cell death rate, whereas PTT alone resulted in a 58% kill rate, while PDT alone resulted in a 40% kill rate. The safety of Mo_2_CT_*x*_ administration was ensured by conducting H&E staining and hematology analysis on blood samples obtained from mice that received intravenous injections of Mo_2_CT_*x*_. From this study, Mo_2_CT_*x*_ has been validated as a dual-purpose agent for both PA imaging and CT imaging, attributed to its photothermal properties and strong X-ray attenuation capabilities.

In another work, W. Feng *et al.* developed ultrathin Mo_2_CT_*x*_ MXenes for efficient photonic tumor hyperthermia which exhibited broad absorption bands that span both NIR-I and NIR-II windows.^70^ They evaluated the biodegradation behaviour of Mo_2_CT_*x*_-PVA nanoflakes under different pH conditions and in physiological solutions, emphasizing their potential for durable therapeutic performance in the tumor microenvironment. The *in vitro* and *in vivo* anticancer activities of Mo_2_CT_*x*_-PVA nanoflakes demonstrated their high PTCE and low cytotoxicity. The computational simulation and experimental validation demonstrated the intense NIR absorption and desirable PTCE of Mo_2_CT_*x*_-PVA nanoflakes, making them promising candidates for efficient photonic cancer hyperthermia.

**Fig. 10:**
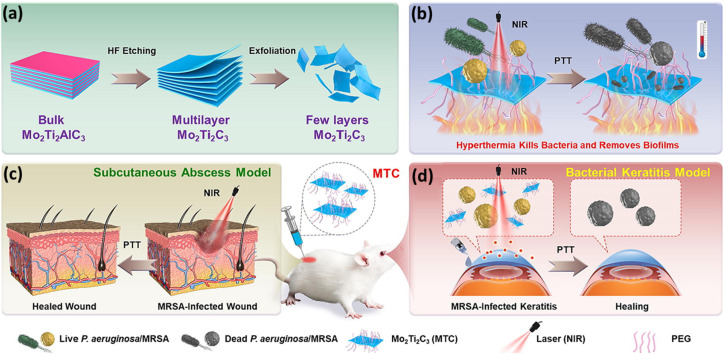
(a) Schematic showing the fabrication process of Mo_2_Ti_2_C_3_T_*x*_. (b–d) Diagram showcasing the action of Mo_2_Ti_2_C_3_T_*x*_ MXenes as PTAs against MRSA to promote MRSA-infected subcutaneous abscess and bacterial keratitis healing through the subcutaneous abscess model and bacterial keratitis model, respectively. Reprinted with permission form ref. 123. Copyright © 2023 The Authors. Published by Elsevier Ltd.

Other than Mo_2_CT_*x*_, mixed-metal MXenes have also been used for hyperthermal-based treatment for wound healing applications. S. Hua *et al.* synthesized Mo_2_Ti_2_C_3_T_*x*_ which shows excellent antibacterial, wound healing, and anti-inflammatory properties.^123^ The MRSA infected subcutaneous of cornea is treated with Mo_2_Ti_2_C_3_T_*x*_ for the prevention of tissue proliferation and tissue inflammations. The combination of Mo_2_Ti_2_C_3_T_*x*_ with NIR successfully disrupted the biofilm structure and eradicated the majority of bacteria within the biofilm. The combination of Mo_2_Ti_2_C_3_T_*x*_ with NIR radiation resulted in extensive wrinkling, shrinkage, and significant damage to the cell membranes of MRSA, indicating synergistic activity between Mo_2_Ti_2_C_3_T_*x*_ and NIR radiation, as shown in Fig. 10. The cytotoxicity tests conducted on human corneal epithelial cells and mouse fibroblasts revealed that Mo_2_Ti_2_C_3_T_*x*_ demonstrated no cytotoxic effects.

## 5. Conclusion and future perspectives

PTT of tumors is efficient compared to conventional methods due to the capability for deep tissue penetration and high selectivity with minimal effects of surrounding normal cells. However, PTT alone is not enough to completely eliminate cancer cells and prevent tumor recurrence and spread. Combining PTT with chemotherapy, PDT or immunotherapy can be a viable alternate approach for treating cancer with high efficiency.

MXenes exhibit desirable and adjustable characteristics, and they have experienced tremendous growth in the field of biomedicine. MXenes have also been known for their ability to absorb light in the NIR region and generate heat by LSPR affects and electron–phonon coupling. This heating effect of MXenes makes them suitable photothermal agents (PTAs), particularly for PTT applications. The adjustable surface of MXenes, along with their high surface area-to-volume ratio, can be employed for the combinatorial approach of PTT along with drug delivery, photodynamic therapy (PDT), bone regeneration and other applications. Even though Ti-based MXenes have been highly investigated, non-Ti MXenes are known to be more biocompatible than Ti MXenes, and hence, they can be the appropriate candidate for different biomedical applications. This review gives an overview of the application of non-Ti MXenes for PTT and its combinatorial approaches for the cancer therapy as well as antibacterial applications.

In order to achieve the desired therapeutic effects in combined therapy, it is crucial to effectively transport both the PTA and therapeutic agents in a synergistic manner. This poses additional challenges for the development of MXene-therapeutic agent composites. Additionally, it is necessary to develop a nanoplatform based on MXenes, which is responsive to the tumor microenvironment. This nanocomposite platform should be targeted to the tumor microenvironment and should be able to respond to changes in pH and temperature, and to various receptors overexpressed on the cancer cells. Hence, the nanocomposite platform would be to strategically deliver drugs and probes, enabling more effective treatment and diagnosis with minimal adverse effects.

**Fig. 11:**
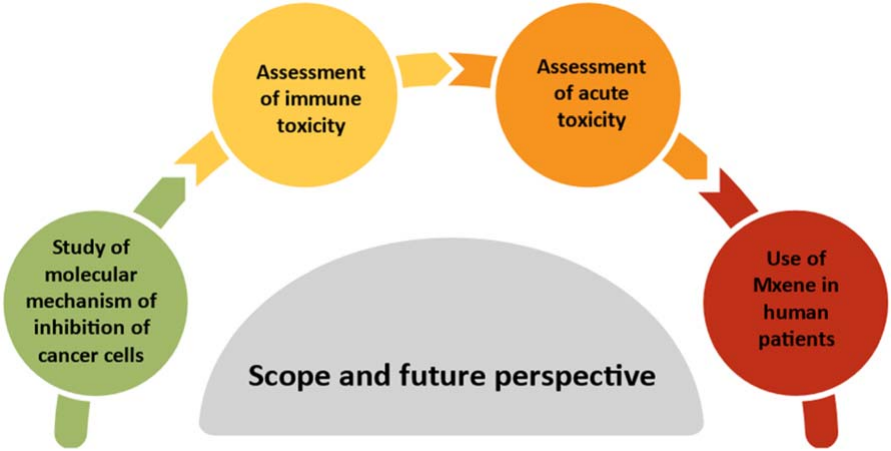
Diagram illustrating the potential scope and future prospects in the realm of photothermal therapy using MXenes.

Another limitation is the lack of discussion on the potential molecular mechanism by which MXene based nanoplatform inhibits cancer progression. Additionally, the immune toxicological assessments of these systems are still in their early stages. Furthermore, the evaluation of acute toxicity of these systems must also be taken into account. While several MXene materials have been subject to preclinical studies to a certain degree, there has been no notable advancement in their clinical translations. The evaluation of their efficacy in cancer treatment is currently carried out using mouse xenograft animal models, which struggle to accurately replicate the diversity and microenvironment of tumors in human patients. Fig. 11 represents the possible scope and future perspective in the field of PTT using MXene-based systems. To summarize, MXene materials for PTT have experienced significant advancements in recent years. Considering the enhanced biocompatibility of non-Ti MXenes, further advancements and breakthroughs in the field of non-Ti MXene-based systems are highly anticipated. Greater emphasis should be placed on the exploration and development of non-Ti MXene-based photothermal applications, as well as their potential in diagnostic imaging, antimicrobial evaluation, biosensing, and biosafety assessment. In this aspect, this review will offer useful information and insights for forthcoming research on non-Ti MXenes for PTT and its combinatorial approaches.

## Data availability

Data will be made available as per request.

## Author contributions

The manuscript was written through contributions of all authors. All authors have given approval to the final version of the manuscript.

## Conflicts of interest

The authors declare no conflicts of interests.

## References

[cit1] Rasool K., Pandey R. P., Rasheed P. A., Buczek S., Gogotsi Y., Mahmoud K. A. (2019). Water treatment and environmental remediation applications of two-dimensional metal carbides (MXenes). Mater. Today.

[cit2] Rahman M., Al Mamun M. S. (2024). Future prospects of MXenes: synthesis, functionalization, properties, and application in field effect transistors. Nanoscale Adv..

[cit3] Huang J., Li Z., Mao Y., Li Z. (2021). Progress and biomedical applications of MXenes. Nano Sel..

[cit4] Gao F., Xue C., Zhang T., Zhang L., Zhu G.-Y., Ou C., Zhang Y.-Z., Dong X. (2023). MXene-Based Functional Platforms for Tumor Therapy. Adv. Mater..

[cit5] Jiao E., Wu K., Liu Y., Lu M., Zhang H., Zheng H., Xu C.-a., Shi J., Lu M. (2021). Robust bioinspired MXene-based flexible films with excellent thermal conductivity and photothermal properties. Compos. Appl. Sci. Manuf..

[cit6] Singh B., Bahadur R., Maske P., Gandhi M., Singh D., Srivastava R. (2023). Preclinical safety assessment of red emissive gold nanocluster conjugated crumpled MXene nanosheets: a dynamic duo for image-guided photothermal therapy. Nanoscale.

[cit7] Han X., Huang J., Lin H., Wang Z., Li P., Chen Y. (2018). 2D Ultrathin MXene-Based Drug-Delivery Nanoplatform for Synergistic Photothermal Ablation and Chemotherapy of Cancer. Adv. Healthcare Mater..

[cit8] Chen R., Kan L., Duan F., He L., Wang M., Cui J., Zhang Z., Zhang Z. (2021). Surface plasmon resonance aptasensor based on niobium carbide MXene quantum dots for nucleocapsid of SARS-CoV-2 detection. Microchim. Acta.

[cit9] Yang X., Zhang C., Deng D., Gu Y., Wang H., Zhong Q. (2022). Multiple Stimuli-Responsive MXene-Based Hydrogel as Intelligent Drug Delivery Carriers for Deep Chronic Wound Healing. Small.

[cit10] Ding R., Zheng H., Zhao X., Xue F., Li P., Xiong J., Chen Z., Liu Z., Peng Q., He X. (2022). Skin inspired multifunctional crumpled Ti3C2Tx MXene/Tissue composite film. Compos. Appl. Sci. Manuf..

[cit11] Ankitha M., Shabana N., Mohan Arjun A., Muhsin P., Abdul Rasheed P. (2023). Ultrasensitive electrochemical detection of dopamine from human serum samples by Nb2CTx-MoS2 hetero structures. Microchem. J..

[cit12] Bhat A., Anwer S., Bhat K. S., Mohideen M. I. H., Liao K., Qurashi A. (2021). Prospects challenges and stability of 2D MXenes for clean energy conversion and storage applications. npj 2D Mater. Appl..

[cit13] Majeed S., Saravanan M., Danish M., Zakariya N. A., Ibrahim M. N. M., Rizvi E. H., NisaAndrabi S. u., Barabadi H., Mohanta Y. K., Mostafavi E. (2023). Bioengineering of green-synthesized TAT peptide-functionalized silver nanoparticles for apoptotic cell-death mediated therapy of breast adenocarcinoma. Talanta.

[cit14] Samadian H., Salami M. S., Jaymand M., Azarnezhad A., Najafi M., Barabadi H., Ahmadi A. (2020). Genotoxicity assessment of carbon-based nanomaterials; Have their unique physicochemical properties made them double-edged swords?. Mutat. Res. Rev. Mutat. Res..

[cit15] Mishra R. K., Sarkar J., Verma K., Chianella I., Goel S., Nezhad H. Y. (2024). Exploring transformative and multifunctional potential of MXenes in 2D materials for next-generation technology. Open Ceram..

[cit16] Phuong N. T. T., Tho L. H., Nguyen T. T., Nam N. N., Trinh K. T. L. (2023). Application of Mxenes and their composites in plasmon-enhanced optical sensors: Review paper. Sens. Actuators, A.

[cit17] Aslam M., Ahmad T., Manzoor M. H., Laiba, Verpoort F. (2023). MXenes as theranostics: Diagnosis and therapy including in vitro and in vivo applications. Appl. Mater. Today.

[cit18] Lim G. P., Soon C. F., Ma N. L., Morsin M., Nayan N., Ahmad M. K., Tee K. S. (2021). Cytotoxicity of MXene-based nanomaterials for biomedical applications: A mini review. Environ. Res..

[cit19] Naguib M., Mashtalir O., Carle J., Presser V., Lu J., Hultman L., Gogotsi Y., Barsoum M. W. (2012). Two-Dimensional Transition Metal Carbides. ACS Nano.

[cit20] Rasheed P. A., Pandey R. P., Banat F., Hasan S. W. (2022). Recent advances in niobium MXenes: Synthesis, properties, and emerging applications. Matter.

[cit21] Zhou J., Dahlqvist M., Björk J., Rosen J. (2023). Atomic Scale Design of MXenes and Their Parent Materials-From Theoretical and Experimental Perspectives. Chem. Rev..

[cit22] Ronchi R. M., Arantes J. T., Santos S. F. (2019). Synthesis, structure, properties and applications of MXenes: Current status and perspectives. Ceram. Int..

[cit23] Tang M., Li J., Wang Y., Han W., Xu S., Lu M., Zhang W., Li H. (2022). Surface Terminations of MXene: Synthesis, Characterization, and Properties. Symmetry.

[cit24] Sokol M., Natu V., Kota S., Barsoum M. W. (2019). On the Chemical Diversity of the MAX Phases. Trends Chem..

[cit25] Dahlqvist M., Barsoum M. W., Rosen J. (2024). MAX phases – Past, present, and future. Mater. Today.

[cit26] Xu B., Gogotsi Y. (2020). MXenes – The fastest growing materials family in the two-dimensional world. Chin. Chem. Lett..

[cit27] VahidMohammadi A., Rosen J., Gogotsi Y. (2021). The world of two-dimensional carbides and nitrides (MXenes). Science.

[cit28] Xu C. , Chen L., Liu Z., Cheng H.-M. and Ren W., Bottom-up synthesis of 2D transition metal carbides and nitrides, in 2D Metal Carbides and Nitrides (MXenes), ed. B. Anasori and Y. Gogotsi, Springer, Cham, 2019, pp. 89–109

[cit29] Pandey R. P., Rasheed P. A., Gomez T., Rasool K., Ponraj J., Prenger K., Naguib M., Mahmoud K. A. (2020). Effect of Sheet Size and Atomic Structure on the Antibacterial Activity of Nb-MXene Nanosheets. ACS Appl. Nano
Mater..

[cit30] Xiao J., Zhao J., Ma X., Li L., Su H., Zhang X., Gao H. (2021). One-step synthesis Nb2CTx MXene with excellent lithium-ion storage capacity. J. Alloys Compd..

[cit31] Halim J., Kota S., Lukatskaya M. R., Naguib M., Zhao M.-Q., Moon E. J., Pitock J., Nanda J., May S. J., Gogotsi Y. (2016). Synthesis and Characterization of 2D Molybdenum Carbide (MXene). Adv. Funct. Mater..

[cit32] Mashtalir O., Naguib M., Mochalin V. N., Dall’Agnese Y., Heon M., Barsoum M. W., Gogotsi Y. (2013). Intercalation and delamination of layered carbides and carbonitrides. Nat. Commun..

[cit33] Naguib M., Unocic R. R., Armstrong B. L., Nanda J. (2015). Large-scale delamination of multi-layers transition metal carbides and carbonitrides "mXenes". Dalton Trans..

[cit34] Mashtalir O., Lukatskaya M. R., Zhao M.-Q., Barsoum M. W., Gogotsi Y. (2015). Amine-Assisted Delamination of Nb2C MXene for Li-Ion Energy Storage Devices. Adv. Mater..

[cit35] Urbankowski P., Anasori B., Makaryan T., Er D., Kota S., Walsh P. L., Zhao M., Shenoy V. B., Barsoum M. W., Gogotsi Y. (2016). Synthesis of two-dimensional titanium nitride Ti4N3 (MXene). Nanoscale.

[cit36] Tran M. H., Schäfer T., Shahraei A., Dürrschnabel M., Molina-Luna L., Kramm U. I., Birkel C. S. (2018). Adding a New Member to the MXene Family: Synthesis, Structure, and Electrocatalytic Activity for the Hydrogen Evolution Reaction of V4C3Tx. ACS Appl. Energy Mater..

[cit37] Anasori B., Xie Y., Beidaghi M., Lu J., Hosler B. C., Hultman L., Kent P. R. C., Gogotsi Y., Barsoum M. W. (2015). Two-Dimensional, Ordered, Double Transition Metals Carbides (MXenes). ACS Nano.

[cit38] Patil A. M., Jadhav A. A., Chodankar N. R., Avatare A. T., Hong J., Dhas S. D., Patil U. M., Jun S. C. (2024). Recent progress of MXene synthesis, properties, microelectrode fabrication techniques for microsupercapacitors and microbatteries energy storage devices and integration: A comprehensive review. Coord. Chem. Rev..

[cit39] Deysher G., Shuck C. E., Hantanasirisakul K., Frey N. C., Foucher A. C., Maleski K., Sarycheva A., Shenoy V. B., Stach E. A., Anasori B. (2020). *et al.*, Synthesis of Mo4VAlC4 MAX Phase and Two-Dimensional Mo4VC4 MXene with Five Atomic Layers of Transition Metals. ACS Nano.

[cit40] Downes M., Shuck C. E., Lord R. W., Anayee M., Shekhirev M., Wang R. J., Hryhorchuk T., Dahlqvist M., Rosen J., Gogotsi Y. (2023). M5X4: A Family of MXenes. ACS Nano.

[cit41] Mei J., Ayoko G. A., Hu C., Bell J. M., Sun Z. (2020). Two-dimensional fluorine-free mesoporous Mo2C MXene via UV-induced selective etching of Mo2Ga2C for energy storage. Sustainable Mater. Technol..

[cit42] Ljubek G., Kralj M., Kraljić Roković M. (2023). Fluorine-free mechanochemical synthesis of MXene. Mater. Sci. Technol..

[cit43] An Y., Tian Y., Man Q., Shen H., Liu C., Xiong S., Feng J. (2023). Fluorine- and Acid-Free Strategy toward Scalable Fabrication of Two-Dimensional MXenes for Sodium-Ion Batteries. Nano Lett..

[cit44] Pang S.-Y., Wong Y.-T., Yuan S., Liu Y., Tsang M.-K., Yang Z., Huang H., Wong W.-T., Hao J. (2019). Universal Strategy for HF-Free Facile and Rapid Synthesis of Two-dimensional MXenes as Multifunctional Energy Materials. J. Am. Chem. Soc..

[cit45] Zada S., Dai W., Kai Z., Lu H., Meng X., Zhang Y., Cheng Y., Yan F., Fu P., Zhang X. (2020). *et al.*, Algae Extraction Controllable Delamination of Vanadium Carbide Nanosheets with Enhanced Near-Infrared Photothermal Performance. Angew. Chem., Int. Ed..

[cit46] Wang D., Zhou C., Filatov A. S., Cho W., Lagunas F., Wang M., Vaikuntanathan S., Liu C., Klie R. F., Talapin D. V. (2023). Direct synthesis and chemical vapor deposition of 2D carbide and nitride MXenes. Science.

[cit47] Xu C., Wang L., Liu Z., Chen L., Guo J., Kang N., Ma X.-L., Cheng H.-M., Ren W. (2015). Large-area high-quality 2D ultrathin Mo2C superconducting crystals. Nat. Mater..

[cit48] Xu C., Song S., Liu Z., Chen L., Wang L., Fan D., Kang N., Ma X., Cheng H.-M., Ren W. (2017). Strongly Coupled High-Quality Graphene/2D Superconducting Mo2C Vertical Heterostructures with Aligned Orientation. ACS Nano.

[cit49] Wang Z., Kochat V., Pandey P., Kashyap S., Chattopadhyay S., Samanta A., Sarkar S., Manimunda P., Zhang X., Asif S. (2017). *et al.*, Metal Immiscibility Route to Synthesis of Ultrathin Carbides, Borides, and Nitrides. Adv. Mater..

[cit50] Joshi S., Wang Q., Puntambekar A., Chakrapani V. (2017). Facile Synthesis of Large Area Two-Dimensional Layers of Transition-Metal Nitride and Their Use as Insertion Electrodes. ACS Energy Lett..

[cit51] Zhang F., Zhang Z., Wang H., Chan C. H., Chan N. Y., Chen X. X., Dai J.-Y. (2017). Plasma-enhanced pulsed-laser deposition of single-crystalline M o 2 C ultrathin superconducting films. Phys. Rev. Mater..

[cit52] Wolden C. A., Pickerell A., Gawai T., Parks S., Hensley J., Way J. D. (2011). Synthesis of β-Mo2C Thin Films. ACS Appl. Mater. Interfaces.

[cit53] Li M., Lu J., Luo K., Li Y., Chang K., Chen K., Zhou J., Rosen J., Hultman L., Eklund P. (2019). *et al.*, Element Replacement Approach by Reaction with Lewis Acidic Molten Salts to Synthesize Nanolaminated MAX Phases and MXenes. J. Am. Chem. Soc..

[cit54] Li Y., Shao H., Lin Z., Lu J., Liu L., Duployer B., Persson P. O. Å., Eklund P., Hultman L., Li M. (2020). *et al.*, A general Lewis acidic etching route for preparing MXenes with enhanced electrochemical performance in non-aqueous electrolyte. Nat. Mater..

[cit55] Siwal S. S., Kaur H., Chauhan G., Thakur V. K. (2023). MXene-Based Nanomaterials for Biomedical Applications: Healthier Substitute Materials for the Future. Adv. NanoBiomed Res..

[cit56] Huang K., Li Z., Lin J., Han G., Huang P. (2018). Two-dimensional transition metal carbides and nitrides (MXenes) for biomedical applications. Chem. Soc. Rev..

[cit57] Szuplewska A., Kulpińska D., Jakubczak M., Dybko A., Chudy M., Olszyna A., Brzózka Z., Jastrzębska A. M. (2022). The 10th anniversary of MXenes: Challenges and prospects for their surface modification toward future biotechnological applications. Adv. Drug Delivery Rev..

[cit58] Yang G., Liu F., Zhao J., Fu L., Gu Y., Qu L., Zhu C., Zhu J.-J., Lin Y. (2023). MXenes-based nanomaterials for biosensing and biomedicine. Coord. Chem. Rev..

[cit59] Xu D., Li Z., Li L., Wang J. (2020). Insights into the Photothermal Conversion of 2D MXene Nanomaterials: Synthesis, Mechanism, and Applications. Adv. Funct. Mater..

[cit60] Lin H., Gao S., Dai C., Chen Y., Shi J. (2017). A Two-Dimensional Biodegradable Niobium Carbide (MXene) for Photothermal Tumor Eradication in NIR-I and NIR-II Biowindows. J. Am. Chem. Soc..

[cit61] Han X., Jing X., Yang D., Lin H., Wang Z., Ran H., Li P., Chen Y. (2018). Therapeutic mesopore construction on 2D Nb(2)C MXenes for targeted and enhanced chemo-photothermal cancer therapy in NIR-II biowindow. Theranostics.

[cit62] Gao L., Chen H., Zhang F., Mei S., Zhang Y., Bao W., Ma C., Yin P., Guo J., Jiang X. (2020). *et al.*, Ultrafast Relaxation Dynamics and Nonlinear Response of Few-Layer Niobium Carbide MXene. Small Methods.

[cit63] Huang Y., Zhou J., Wang G., Sun Z. (2019). Abnormally Strong Electron–Phonon Scattering Induced Unprecedented Reduction in Lattice Thermal Conductivity of Two-Dimensional Nb2C. J. Am. Chem. Soc..

[cit64] Venkateshalu S., Shariq M., Kim B., Patel M., Mahabari K. S., Choi S.-I., Chaudhari N. K., Grace A. N., Lee K. (2023). Recent advances in MXenes: beyond Ti-only systems. J. Mater. Chem. A.

[cit65] Wen Y., Hu L., Li J., Geng Y., Yang Y., Wang J., Chen X., Yu L., Tang H., Han T. (2022). *et al.*, Exposure to two-dimensional ultrathin Ti3C2 (MXene) nanosheets during early pregnancy impairs neurodevelopment of offspring in mice. J. Nanobiotechnol..

[cit66] Wei Y., Bao R., Hu L., Geng Y., Chen X., Wen Y., Wang Y., Qin M., Zhang Y., Liu X. (2023). Ti3C2 (MXene) nanosheets disrupt spermatogenesis in male mice mediated by the ATM/p53 signaling pathway. Biol. Direct.

[cit67] Fusco L., Gazzi A., Shuck C. E., Orecchioni M., Alberti D., D'Almeida S. M., Rinchai D., Ahmed E., Elhanani O., Rauner M. (2022). *et al.*, Immune Profiling and Multiplexed Label-Free Detection of 2D MXenes by Mass Cytometry and High-Dimensional Imaging. Adv. Mater..

[cit68] Yang G., Zhao J., Yi S., Wan X., Tang J. (2020). Biodegradable and photostable Nb2C MXene quantum dots as promising nanofluorophores for metal ions sensing and fluorescence imaging. Sens. Actuators, B.

[cit69] Gu M., Dai Z., Yan X., Ma J., Niu Y., Lan W., Wang X., Xu Q. (2021). Comparison of toxicity of Ti3C2 and Nb2C Mxene quantum dots (QDs) to human umbilical vein endothelial cells. J. Appl. Toxicol..

[cit70] Feng W., Wang R., Zhou Y., Ding L., Gao X., Zhou B., Hu P., Chen Y. (2019). Ultrathin Molybdenum Carbide MXene with Fast Biodegradability for Highly Efficient Theory-Oriented Photonic Tumor Hyperthermia. Adv. Funct. Mater..

[cit71] Fusco L., Gazzi A., Shuck C. E., Orecchioni M., Ahmed E. I., Giro L., Zavan B., Yilmazer A., Ley K., Bedognetti D. (2023). *et al.*, V4C3 MXene Immune Profiling and Modulation of T Cell-Dendritic Cell Function and Interaction. Small Methods.

[cit72] Ren X., Huo M., Wang M., Lin H., Zhang X., Yin J., Chen Y., Chen H. (2019). Highly Catalytic Niobium Carbide (MXene) Promotes Hematopoietic Recovery after Radiation by Free Radical Scavenging. ACS Nano.

[cit73] Maleski K., Shuck C. E., Fafarman A. T., Gogotsi Y. (2021). The Broad Chromatic Range of Two-Dimensional Transition Metal Carbides. Adv. Opt. Mater..

[cit74] Feng G., Zhang G.-Q., Ding D. (2020). Design of superior phototheranostic agents guided by Jablonski diagrams. Chem. Soc. Rev..

[cit75] Husni P., Shin Y., Kim J. C., Kang K., Lee E. S., Youn Y. S., Rusdiana T., Oh K. T. (2020). Photo-Based Nanomedicines Using Polymeric Systems in the Field of Cancer Imaging and Therapy. Biomedicines.

[cit76] Planner A., Frąckowiak D. (2001). Fast and slow processes of thermal deactivation of excited stilbazolium merocyanine dyes. J. Photochem. Photobiol., A.

[cit77] Gao D., Guo X., Zhang X., Chen S., Wang Y., Chen T., Huang G., Gao Y., Tian Z., Yang Z. (2020). Multifunctional phototheranostic nanomedicine for cancer imaging and treatment. Mater. Today Bio.

[cit78] Chen C., Ou H., Liu R., Ding D. (2020). Regulating the Photophysical Property of Organic/Polymer Optical Agents for Promoted Cancer Phototheranostics. Adv. Mater..

[cit79] Vankayala R., Hwang K. C. (2018). Near-Infrared-Light-Activatable Nanomaterial-Mediated Phototheranostic Nanomedicines: An Emerging Paradigm for Cancer Treatment. Adv. Mater..

[cit80] Hao S., Han H., Yang Z., Chen M., Jiang Y., Lu G., Dong L., Wen H., Li H., Liu J. (2022). *et al.*, Recent Advancements on Photothermal Conversion and Antibacterial Applications over MXenes-Based Materials. Nano-Micro Lett..

[cit81] Brochado A. R., Telzerow A., Bobonis J., Banzhaf M., Mateus A., Selkrig J., Huth E., Bassler S., Zamarreño Beas J., Zietek M. (2018). *et al.*, Species-specific activity of antibacterial drug combinations. Nature.

[cit82] Chen H., Wu L., Wang T., Zhang F., Song J., Fu J., Kong X., Shi J. (2023). PTT/PDT-induced microbial apoptosis and wound healing depend on immune activation and macrophage phenotype transformation. Acta Biomater..

[cit83] Larina I. V., Larin K. V., Esenaliev R. O. (2005). Real-time optoacoustic monitoring of temperature in tissues. J. Phys. D: Appl. Phys..

[cit84] Das D., Sharma A., Rajendran P., Pramanik M. (2021). Another decade of photoacoustic imaging. Phys. Med. Biol..

[cit85] Lin L., Wang L. V. (2022). The emerging role of photoacoustic imaging in clinical oncology. Nat. Rev. Clin. Oncol..

[cit86] Richard B., Shahana C., Vivek R., M A. R., Rasheed P. A. (2023). Acoustic platforms meet MXenes – a new paradigm shift in the palette of biomedical applications. Nanoscale.

[cit87] Li G., Lian S., Wang J., Xie G., Zhang N., Xie X. (2023). Surface chemistry engineering and the applications of MXenes. J. Materiomics.

[cit88] Kumar S., Kumari N., Singh T., Seo Y. (2024). Shielding 2D MXenes against oxidative degradation: recent advances, factors and preventive measures. J. Mater. Chem. C.

[cit89] Mozafari M., Soroush M. (2021). Surface functionalization of MXenes. Mater. Adv..

[cit90] Lin S., Lin H., Yang M., Ge M., Chen Y., Zhu Y. (2020). A two-dimensional MXene potentiates a therapeutic microneedle patch for photonic implantable medicine in the second NIR biowindow. Nanoscale.

[cit91] Yin J., Pan S., Guo X., Gao Y., Zhu D., Yang Q., Gao J., Zhang C., Chen Y. (2021). Nb2C MXene-Functionalized Scaffolds Enables Osteosarcoma Phototherapy and Angiogenesis/Osteogenesis of Bone Defects. Nano-Micro Lett..

[cit92] Yang K., Zhao S., Li B., Wang B., Lan M., Song X. (2022). Low temperature photothermal therapy: Advances and perspectives. Coord. Chem. Rev..

[cit93] Li M., Peng X., Han Y., Fan L., Liu Z., Guo Y. (2021). Ti3C2 MXenes with intrinsic peroxidase-like activity for label-free and colorimetric sensing of proteins. Microchem. J..

[cit94] Jin Z., Xu G., Niu Y., Ding X., Han Y., Kong W., Fang Y., Niu H., Xu Y. (2020). Ti3C2Tx MXene-derived TiO2/C-QDs as oxidase mimics for the efficient diagnosis of glutathione in human serum. J. Mater. Chem. B.

[cit95] Yang H., Xia L., Ye X., Xu J., Liu T., Wang L., Zhang S., Feng W., Du D., Chen Y. (2023). Ultrathin Niobium Carbide MXenzyme for Remedying Hypertension by Antioxidative and Neuroprotective Actions. Angew. Chem., Int. Ed..

[cit96] Geng B., Yan L., Zhu Y., Shi W., Wang H., Mao J., Ren L., Zhang J., Tian Y., Gao F. (2023). *et al.*, Carbon Dot@MXene Nanozymes with Triple Enzyme-Mimic Activities for Mild NIR-II Photothermal-Amplified Nanocatalytic Therapy. Adv. Healthcare Mater..

[cit97] Sun X., He X., Zhu Y., Obeng E., Zeng B., Deng H., Shen J., Hu R. (2023). Valence-switchable and biocatalytic vanadium-based MXene nanoplatform with photothermal-enhanced dual enzyme-like activities for anti-infective therapy. Chem. Eng. J..

[cit98] Yang C., Luo Y., Lin H., Ge M., Shi J., Zhang X. (2021). Niobium Carbide MXene Augmented Medical Implant Elicits Bacterial Infection Elimination and Tissue Regeneration. ACS Nano.

[cit99] Han X., Jing X., Yang D., Lin H., Wang Z., Ran H., Li P., Chen Y. (2018). Therapeutic mesopore construction on 2D Nb_2_C MXenes for targeted and enhanced chemo-photothermal cancer therapy in NIR-II biowindow. Theranostics.

[cit100] Lin X., Li Z., Du S., Wang Q., Guan Y., Cheng G., Hong H., Li J., Chen X., Chen T. (2023). Occam's Razor-Inspired Nb2C delivery platform potentiates breast cancer therapy and inhibits lung metastasis. Chem. Eng. J..

[cit101] Hao Z., Li Y., Liu X., Jiang T., He Y., Zhang X., Cong C., Wang D., Liu Z., Gao D. (2021). Enhancing biocatalysis of a MXene-based biomimetic plasmonic assembly for targeted cancer treatments in NIR-II biowindow. Chem. Eng. J..

[cit102] Lu Y., Zhang X., Hou X., Feng M., Cao Z., Liu J. (2021). Functionalized 2D Nb2C nanosheets for primary and recurrent cancer photothermal/immune-therapy in the NIR-II biowindow. Nanoscale.

[cit103] Zhou B., Pu Y., Lin H., Yue W., Yin H., Yin Y., Ren W., Zhao C., Chen Y., Xu H. (2020). In situ phase-changeable 2D MXene/zein bio-injection for shear wave elastography-guided tumor ablation in NIR-II bio-window. J. Mater. Chem. B.

[cit104] Yin J., Pan S., Guo X., Gao Y., Zhu D., Yang Q., Gao J., Zhang C., Chen Y. (2021). Nb(2)C MXene-Functionalized Scaffolds Enables Osteosarcoma Phototherapy and Angiogenesis/Osteogenesis of Bone Defects. Nano-Micro Lett..

[cit105] Yang Q., Yin H., Xu T., Zhu D., Yin J., Chen Y., Yu X., Gao J., Zhang C., Chen Y. (2020). *et al.*, Engineering 2D Mesoporous Silica@MXene-Integrated 3D-Printing Scaffolds for Combinatory Osteosarcoma Therapy and NO-Augmented Bone Regeneration. Small.

[cit106] He C., Yu L., Yao H., Chen Y., Hao Y. (2021). Combinatorial Photothermal 3D-Printing Scaffold and Checkpoint Blockade Inhibits Growth/Metastasis of Breast Cancer to Bone and Accelerates Osteogenesis. Adv. Funct. Mater..

[cit107] Yuan H., Hong X., Ma H., Fu C., Guan Y., Huang W., Ma J., Xia P., Cao M., Zheng L. (2023). *et al.*, MXene-Based Dual Functional Nanocomposite with Photothermal Nanozyme Catalytic Activity to Fight Bacterial Infections. ACS Mater. Lett..

[cit108] Chen J., Liu Y., Cheng G., Guo J., Du S., Qiu J., Wang C., Li C., Yang X., Chen T. (2022). *et al.*, Tailored Hydrogel Delivering Niobium Carbide Boosts ROS-Scavenging and Antimicrobial Activities for Diabetic Wound Healing. Small.

[cit109] Kwon S.-y., Lee J., Jhon Y. I., Lim G., Jhon Y. M., Lee J. H. (2022). Photothermal property investigation of V2CTx MXene and its use for all-optical modulator. Opt. Mater..

[cit110] Wu S., Du S., Guan Y., Lin Q., Liu Y., Lv R., Zhang Z., Xia Y., Chen T., Hong H. (2023). V2C-Driven Nanodelivery Platform Potentiates Synergistic Breast Cancer Therapy. ACS Mater. Lett..

[cit111] Cao Y., Wu T., Zhang K., Meng X., Dai W., Wang D., Dong H., Zhang X. (2019). Engineered Exosome-Mediated Near-Infrared-II Region V2C Quantum Dot Delivery for Nucleus-Target Low-Temperature Photothermal Therapy. ACS Nano.

[cit112] Zada S., Lu H., Yang F., Zhang Y., Cheng Y., Tang S., Wei W., Qiao Y., Fu P., Dong H. (2021). *et al.*, V2C Nanosheets as Dual-Functional Antibacterial Agents. ACS Appl. Bio Mater..

[cit113] Deng J., Xian D., Cai X., Liao S., Lei S., Han F., An Y., He Q., Quan G., Wu C. (2023). *et al.*, Surface-Engineered Vanadium Carbide MXenzyme for Anti-Inflammation and Photoenhanced Antitumor Therapy of Colon Diseases. Adv. Funct. Mater..

[cit114] Zhao R., Zhu Y., Feng L., Liu B., Hu Y., Zhu H., Zhao Z., Ding H., Gai S., Yang P. (2024). Architecture of Vanadium-Based MXene Dysregulating Tumor Redox Homeostasis for Amplified Nanozyme Catalytic/Photothermal Therapy. Adv. Mater..

[cit115] Zhao W., Jiang L., Yang H., Yu Z., Yang Z., Zhou Y. (2023). Antibacterial effect and photothermal sterilization of low dose two-dimensional vanadium carbide. Appl. Phys. A.

[cit116] Feng X., Xian D., Fu J., Luo R., Wang W., Zheng Y., He Q., Ouyang Z., Fang S., Zhang W. (2023). *et al.*, Four-armed host-defense peptidomimetics-augmented vanadium carbide MXene-based microneedle array for efficient photo-excited bacteria-killing. Chem. Eng. J..

[cit117] Liu Y., He X., Feng J., Wang D., Obeng E., Yu C., Song Y., Shen J., Li Z. (2023). Engineering a new member of MXenes M5C4 phases nanoplatforms as synergistically photothermal and chemodynamic therapeutics for methicillin-resistant Staphylococcus aureus. Chem. Eng. J..

[cit118] Liu Z., Lin H., Zhao M., Dai C., Zhang S., Peng W., Chen Y. (2018). 2D Superparamagnetic Tantalum Carbide Composite MXenes for Efficient Breast-Cancer Theranostics. Theranostics.

[cit119] Lin H., Wang Y., Gao S., Chen Y., Shi J. (2018). Theranostic 2D Tantalum Carbide (MXene). Adv. Mater..

[cit120] Dai C., Chen Y., Jing X., Xiang L., Yang D., Lin H., Liu Z., Han X., Wu R. (2017). Two-Dimensional Tantalum Carbide (MXenes) Composite Nanosheets for Multiple Imaging-Guided Photothermal Tumor Ablation. ACS Nano.

[cit121] Lin P., Luo P., Guo Y., Qiu H., Wang M., Huang G. (2021). Tantalum Nitride Nanosheets for Photoacoustic Imaging-Guided Photothermal Cancer Therapy. Part. Part. Syst. Char..

[cit122] Zhang Q., Huang W., Yang C., Wang F., Song C., Gao Y., Qiu Y., Yan M., Yang B., Guo C. (2019). The theranostic nanoagent Mo2C for multi-modal imaging-guided cancer synergistic phototherapy. Biomater. Sci..

[cit123] Hua S., Huang B., Le Z., Huang Q. (2023). Mo-based Mo2Ti2C3 MXene as photothermal nanoagents to eradicating methicillin-resistant Staphylococcus aureus with photothermal therapy. Mater. Des..

